# Efficient extravasation of tumor-repopulating cells depends on cell deformability

**DOI:** 10.1038/srep19304

**Published:** 2016-01-20

**Authors:** Junjian Chen, Wenwen Zhou, Qiong Jia, Junwei Chen, Shuang Zhang, Wenting Yao, Fuxiang Wei, Yuejin Zhang, Fang Yang, Wei Huang, Yao Zhang, Huafeng Zhang, Yi Zhang, Bo Huang, Zhihong Zhang, Haibo Jia, Ning Wang

**Affiliations:** 1 Laboratory for Cellular Biomechanics and Regenerative Medicine, Department of Biomedical Engineering, School of Life Sciences, Huazhong University of Science and Technology, Wuhan, Hubei 430074, China; 2 Department of Biochemistry and Molecular Biology, Tongji Medical College, Huazhong University of Science and Technology, Wuhan, Hubei 430030 China; 3 Department of Immunology, Institute of Basic Medical Sciences of Chinese Academy of Medical Sciences, Beijing 100005 China; 4 Britton Chance Center for Biomedical Photonics, Wuhan National Laboratory for Optoelectronics-Huazhong University of Science and Technology, Wuhan, Hubei 430074, China; 5 Department of Mechanical Science and Engineering, College of Engineering, University of Illinois at Urbana-Champaign, Urbana, IL 61801 USA

## Abstract

Cancer metastasis is the most deadly stage in cancer progression. Despite significant efforts over the past decades, it remains elusive why only a very small fraction of cancer cells is able to generate micrometastasis and metastatic colonization. Recently we have shown that tumor-repopulating cells (TRCs), a highly tumorigenic subpopulation of mouse melanoma cells, can be selected by being cultured and grown in 3D soft fibrin gels. Here we show that when injected into the yolk of a 2 day-post-fertilization (dpf) embryo of Tg (fli1:EGFP or kdrl:mCherry) zebrafish, TRCs are much more efficient in surviving and growing at various secondary sites to generate micrometastasis and metastatic colonization than control melanoma cells that are grown on rigid plastic. The metastasis of TRCs is dependent on the presence of *Sox2*, a self-renewal gene, and silencing *Sox2* leads to the inhibition of TRC metastasis. High-resolution of 3D confocal images of the TRCs at the secondary sites show that extravasation and formation of micrometastases by TRCs are more efficient than by the control cells. Remarkably, efficient extravasation of TRCs *in vivo* and transmigration *in vitro* are determined by TRC deformability, as a result of low Cdc42 and high Sox2. Our findings suggest that tumor cell deformability is a key factor in controlling extravasation dynamics during metastasis.

Cancer metastasis is the most devastating stage of cancer^1^. Much efforts over the years have been devoted to understanding the process of metastasis^2^,^3^,^4^,^5^,^6^,^7^,^8^,^9^,^10^,^11^ but the underlying mechanisms remain elusive. We recently have shown that a small subpopulation of melanoma tumor cells, selected from a general population of B16 melanoma cells and grown in a soft 3D fibrin matrix, are highly tumorigenic in culture and in syngeneic and nonsyngeneic immunocompetent mice^12^,^13^. These cells are defined as tumor-repopulating cells (TRCs)^13^. We have shown that the self-renewal of these TRCs depend on the expression of Sox2 in mice^13^. However, mouse tissues are opaque and thus are not appropriate for visualization and quantification of the dynamic processes of tumor cell metastasis. In previously published reports zebrafish has been used as a useful vertebrate model to study metastatic processes of tumors^11^,^14^,^15^,^16^. In this study, we used transparent zebrafish Tg(fli1:EGFP) or Tg(kdr1:mCherry) to image metastatic processes with high-resolution microscopy after mouse melanoma B16 cells expressing KatushkaS158A, a tetrameric far-red fluorescent protein (tfRFP)^17^, or B16 cells transfected with YFP (yellow fluorescent protein) or CFP (cyan fluorescent protein), are injected into the yolk (or pericardium cavity) of the developing zebrafish 2 days post fertilization (2 dpf). We quantified extravasation dynamics of these tumor cells in zebrafish at various times post injection.

## Results

### TRCs are more proliferative and metastatic in zebrafish.

**Figure 1:**
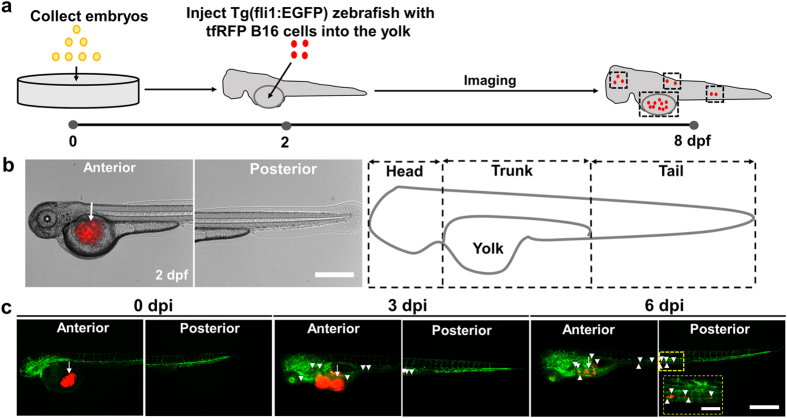
Establishment of a zebrafish model to quantify mouse melanoma cell behaviors *in vivo*. (**a**) Schematic of experimental protocols. (**b**) Left panels: Representative images show that 500 tfRFP B16 cells were injected into the yolk sac (white arrow) of a zebrafish. Right panels: the fish was divided into three main sections (Head, Trunk and Tail) to quantitate metastatic sites of the tumor cells. Scale bar, 500 μm. (**c**) Approximately five hundred tfRFP B16 cells were injected into the yolk sac of 2 day post-fertilization (2 dpf) embryos. Left, middle, and right panels: fluorescent images of the fish at 0, 3, and 6 dpi. Images are representative of >100 injected embryos. Arrowheads indicate disseminated tumor foci (single tumor cells or cell aggregates). Color code: in (**a**), embryos are yellow, mouse melanoma cells are red; in (**c**), zebrafish blood vessels are green and melanoma cells are red. Scale bar, 500 μm. Inset: yellow-dashed line rectangle box is the enlarged image of the small yellow box above; scale bar, 100 μm.

To visualize tumor cell metastasis *in vivo*, we used Tg (fli1:EGFP)^18^,^19^ that is a line of transgenic zebrafish in which the whole blood vessel system throughout embryogenesis is fluorescent. Single mouse melanoma tfRFP-B16 cells were injected into the yolk sac of the embryo 2 days post-fertilization (2 dpf or 0 dpi (day post-injection)) (Fig. 1a,b). To visualize tumor cell metastasis at an early embryonic stage of zebrafish, we imaged the transgenic zebrafish daily under fluorescent microscopy from 2 dpf to 8 dpf without causing any apparent adverse effects to the fish development. The anterior and posterior parts of the fish (Fig. 1b left two panels, 1c) were subdivided into three sections: head, trunk, and tail in order to better distinguish locations of metastatic tumor foci (Fig. 1b right). To quantify temporal and spatial changes of the tumor size in the fish after injection, we calibrated the red fluorescence intensity and area of the injected tfRFP melanoma cells in the fish yolk at 0 dpi (Supplementary Fig. 1). The number of the tumor cells increased at the primary site (yolk) from 0 dpi (~500 cells being injected) to 3 dpi (Fig. 1c, left panels, middle panels) and then suddenly decreased at 4 dpi (Fig. 1c, right panels), likely due to the removal of the melanoma cells after initiation of the zebrafish immune system after ~5 dpf (3 dpi)^20^,^21^,^22^,^23^. However, at 6 dpi (8 dpf), some tumor cells still survived at the primary site while others continued to metastasize towards the tail (Fig. 1c right panels). Treatment with dexamethasone (Dex), a known immune suppressor^15^, prevented the sudden drop in the tumor size between 3 dpi and 4 dpi and led to survival of more tumor cells at 5 dpi and 6 dpi (Supplementary Fig. 2), supporting the notion that the activation of the immune system was responsible for the sudden drop in tumor size between 3 and 4 pi.

To determine if this fish model is a model of metastasis relevant to cancer or one of simple cell distribution throughout the zebrafish, we compared non-cancerous mouse CFP-3T3 cells cultured on rigid plastic with YFP-B16-F1 cells cultured on rigid plastic after co-injecting at 1:1 ratio into the pericardium cavity of Tg (kdrl:mCherry) zebrafish. We found that these non-cancerous 3T3 cells extravasated much less than B16-F1 melanoma cells: at 12 hpi, only 6% of the 3T3 cells at the tail extravasated, compared with 12.9% of the B16-F1 melanoma cells at the tail; at 24 hpi, 12.2% for 3T3 cells vs 21.6% for B16-F1 melanoma cells (Supplementary Fig. 3). These findings suggest that the zebrafish could be used as a model of extravasation relevant to cancer metastasis.

**Figure 2:**
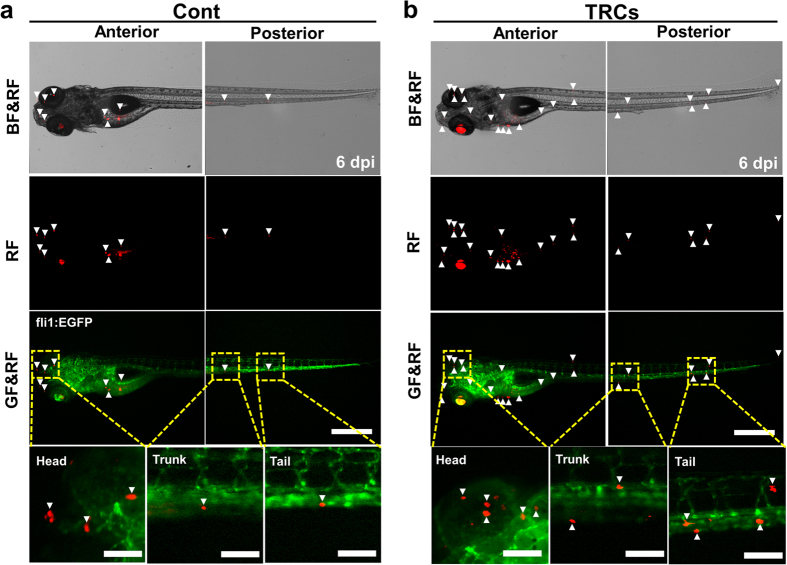
TRCs exhibit more metastases. The same number (~500 cells) of tfRFP melanoma cells cultured on 2D rigid plastic for 5 days (**Cont**) or in 3D 90-Pa fibrin gels for 5 days (TRCs) was injected into the yolk of 2 dpf embryos respectively. Representative images show metastatic tumor foci at 6 dpi zebrafish, comparing the **TRCs** group (**b**) with the **Cont** group (**a**). Arrowheads point to disseminated tumor foci (single tumor cells or tumor cell aggregates). There are more arrowheads in the TRCs group, suggesting more metastases. Color code: Zebrafish blood vessels are green, and melanoma cells are red; BF = brightfield; GF = Green Fluorescence; RF = tfRFP Fluorescence. Scale bars, 500 μm.

**Figure 3:**
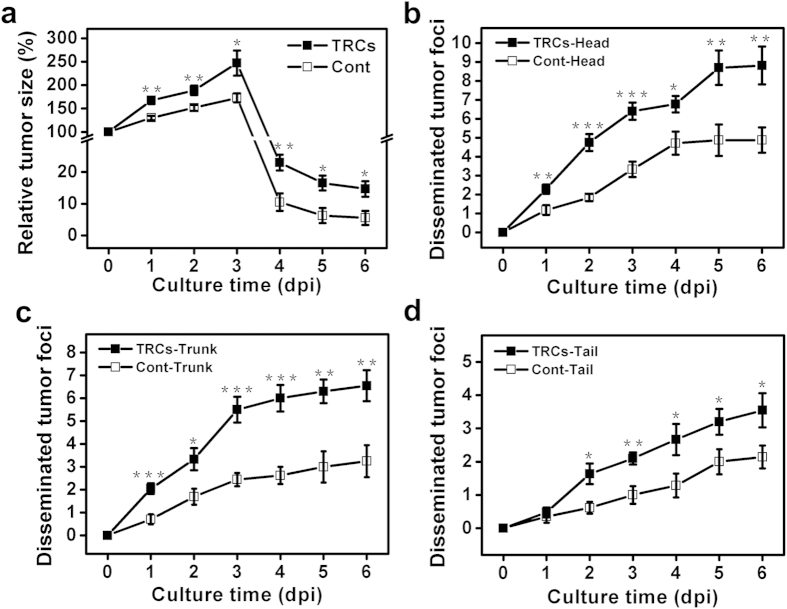
TRCs are more proliferative and metastatic in zebrafish. (**a**) Quantification of total tumor sizes (from primary and secondary sites) from 0 dpi to 6 dpi relative to the injected tumor size at the yolk (n >10 fish per group, ≥3 independent experiments). (**b, c, d**) Quantification of tumor cell metastasis from 0 dpi to 6 dpi at Head (**b**), Trunk (**c**) and Tail (**d**) (n >10 fish per group, ≥3 independent experiments). Mean + s.e.m.; *p < 0.05;**p < 0.01, ***p < 0.001.

To elucidate how melanoma TRCs proliferate and metastasize *in vivo*, we mechanically selected TRCs by culturing tfRFP-B16 in 3D soft fibrin gels (~90 Pa) for 5 days^12^. These cells were then gently sucked out of the soft gel with a pipette, re-suspended as single cells in medium, counted, and injected into the yolk sac of the Tg (flil1:egfp) embryos at 2 dpf. Proliferation, survival, and metastasis of these TRCs were compared with those of control cells cultured on rigid plastic (Cont) from 0 dpi to 6 dpi (Fig. 2; Supplementary Figs 4 and 5). From 1 dpi to 3dpi, the tumor size of TRCs increased more rapidly (to ~250%) than that of Cont (to ~170%) (Fig. 3a), consistent with high self-renew ability of TRCs that express self-renewal gene Sox2 12,13. From 4 dpi to 6 dpi, the size of the tumor cells decreased dramatically due to the initiation of the fish immune system^20^,^21^,^22^,^23^, but TRCs survived better than Cont, consistent with the previously published results in mice^12^. Interestingly, the number of disseminated tumor foci in all three parts (Head, Trunk, and Tail) of the fish body were greater in TRC-injected fish than Cont-injected fish from 2 dpi through 6 dpi, suggesting that TRCs are more metastatic than control melanoma cells (Fig. 3b–d). Subdividing the fish body into a few more body parts, we found that a higher percentage of fish larvae injected with TRCs had metastases in the head, the liver, the pancreas, and the tail than that injected with Cont (Supplementary Fig. 6). In contrast, a similar percentage of larvae injected with TRCs had metastases in the heart, the dorsal section, and the intestine when compared with Cont, possibly due to similar extravasation dynamics to those body parts. We noticed that more metastases were observed at the head and the trunk of TRC-injected fish as early as 1 dpi, while the difference at the tail appeared only at 2 dpi (Fig. 3b–d). Since blood carries tumor cells to different parts of the fish body very quickly (on the order of seconds), the difference in the timing of metastasis for TRC verses Cont between the tail and the head (or the trunk) cannot be explained by the distance from the yolk to the tail, suggesting that dynamic processes of extravasation might be different in different tissues.

### Sox2 regulates tumor cell metastasis *in vivo*.

**Figure 4:**
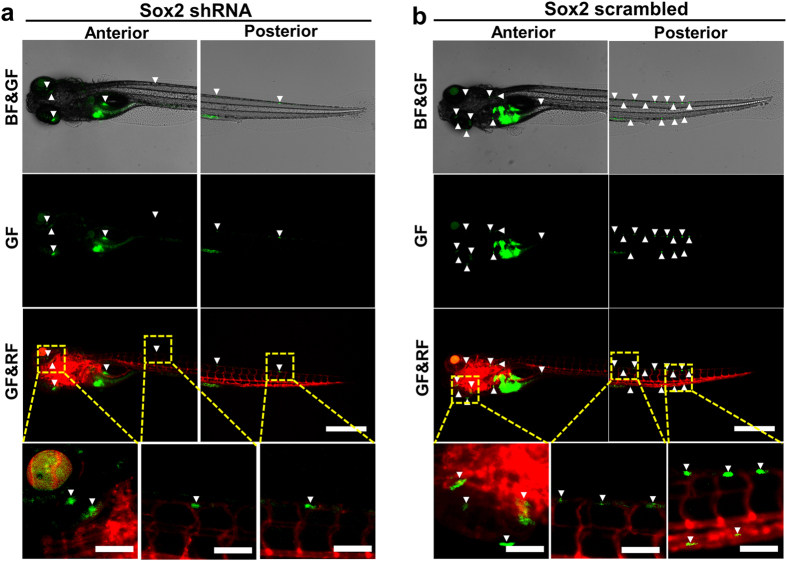
Silencing Sox2 inhibits metastasis of TRCs. Sox2 shRNA (**a**) and scrambled shRNA (**b**) treated TRCs were injected into the yolk of 2 dpf Tg(kdrl:mCherry) embryos respectively; cell proliferation, survival, and metastasis were quantified every 24 hr from 0 dpi to 6 dpi respectively. Representative images show metastatic tumor foci at 6 dpi zebrafish by comparing the scrambled shRNA group with the Sox2 shRNA group. Arrowheads indicate disseminated tumor foci or tumor foci aggregates. Color code: Sox2 shRNA or scrambled shRNA treated TRCs are green; zebrafish blood vessels are red. Scale bars, 500 μm.

**Figure 5:**
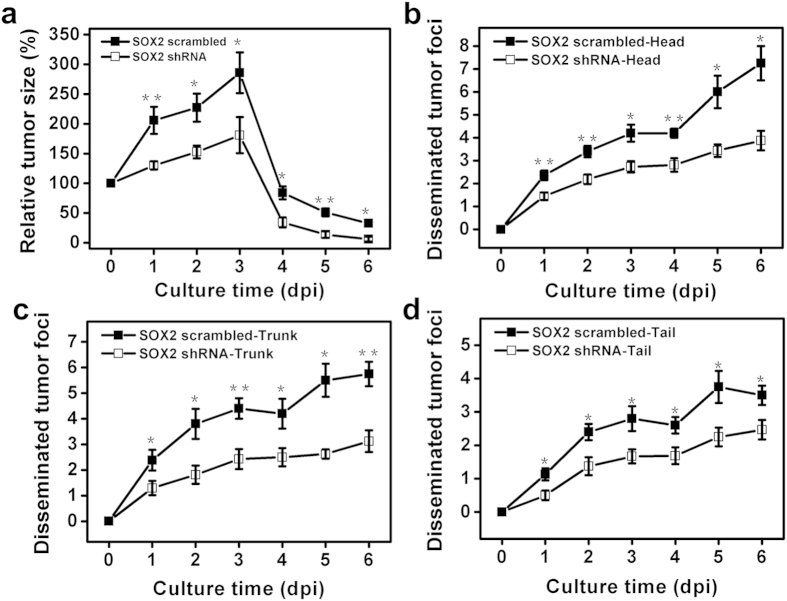
Silencing Sox2 downregulates proliferation and metastases of TRCs in zebrafish. (**a**) Quantification of total tumor sizes from 0 dpi to 6 dpi relative to the injected tumor size at the yolk (n >10 fish per group; ≥3 independent experiments). (**b–d**) Quantification of tumor cell metastasis from 0 dpi to 6 dpi at Head (**b**), Trunk (**c**), and Tail (**d**) (n >10 fish per group, ≥3 independent experiments). Mean + s.e.m.; *p < 0.05, **p < 0.01.

Sox2 is highly expressed in TRCs and is essential in self-renewal of the TRCs^12^,^13^. To determine the impact of Sox2 in dynamics processes of metastasis *in vivo*, we silenced *Sox2* in TRCs via shRNA interference and then compared the *Sox2* shRNA treated group with the scrambled control group. Since both *Sox2* shRNA treated TRCs and scrambled shRNA treated TRCs emitted green fluorescence, we had to use Tg(kdrl:mCherry) zebrafish to visualize blood vessels (red color) and shRNA transfected tumor cell (green color) proliferation and metastasis simultaneously. Silencing *Sox2* in TRCs significantly decreased the size of the primary tumor and the number of disseminated tumor foci when compared with scrambled control (Fig. 4); summarized data show that tumor sizes were much smaller in the *Sox2* shRNA group than the scrambled group from 1 dpi through 6 dpi (Fig. 5a), suggesting that Sox2 is essential in cell self-renewal and survival, extending previously published results in mice^12^,^13^. Quantification of disseminated tumor foci in Head, Trunk, and Tail show that there were fewer foci after silencing *Sox2* of TRCs than after treatment with scrambled control (Fig. 5c–e). Comparing these data in Fig. 5 with those in Fig. 3 reveal that *Sox2* shRNA treated TRCs behaved quantitatively similar to those untreated melanoma cells grown on 2D rigid plastic (Supplementary Fig. 7), further strengthening the finding that Sox2 is critical in the dynamic processes of metastasis *in vivo* by melanoma cells, in addition to its essential roles in self-renewal^12^,^13^.

### Mechanism of efficient extravasation by TRCs

**Figure 6:**
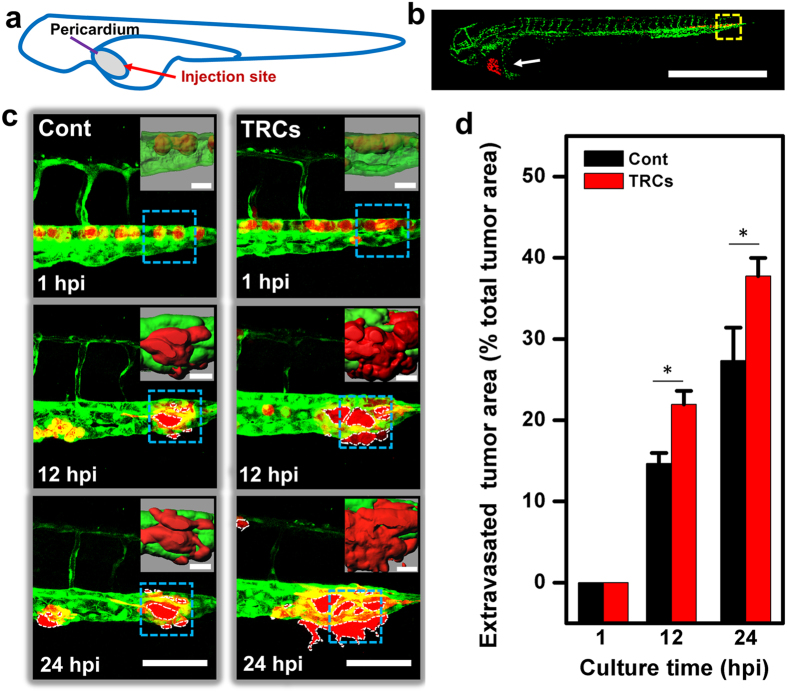
TRCs extravasate more effectively than control melanoma cells *in vivo*. (**a**) Schematic of injecting tumor cells into the pericardium of the fish. (**b**) A low resolution confocal image showing injected tumor cells (white arrow) being moved to the tail (yellow dashed-line box) 1 hr after injection. Scale bar, 1 mm. (**c**) TRCs and Cont were injected into the pericardium of 48 hpf embryos respectively. Images in each panel (246 μm × 246 μm) corresponding to the yellow dashed-line box area in (**b**) were acquired with high resolution confocal microscopy (63 × objective), showing vessel penetration of Cont (left panels) or TRCs (right panels) at 1 (yet to penetrate), 12 and 24 hpi respectively and summarized in (**d**). Acquired images were stacked in z-axis and vessel boundaries were outlined to quantify the cell areas that were out of the vessels as extravasated areas and that were inside the vessels. Dashed white lines mark the tumor extravasated areas (i.e., various sizes of micrometastases) from vessels to surrounding tissues. Insets were 3D-images reconstructed using Imaris software that correspond to respective blue dashed boxes, showing tumor cells stay inside the vessels at 1 hpi and extravasated gradually at 12 and 24 hpi. Scale bars, 100 μm; Insets scale bars, 20 μm. It is of interest to note that the extravasated cells are in general cell aggregates. (**d**) Quantification of extravasated tumor area relative to the total tumor area at different time points: 1, 12, and 24 hpi. Color code: Zebrafish blood vessels are green, and mouse tumor cells are red. TRCs exhibit higher penetration rates than Cont. (n >6 fish per group; ≥3 independent experiments). Mean + s.e.m.; *p < 0.05.

To better understand how TRCs metastasize, we need to examine early time points of the dynamics of TRCs compared with control melanoma cells. However, at 1 dpi (24 hours post injection (hpi)), there were no differences for disseminated tumor foci between TRCs and control melanoma cells (Fig. 3d) when the tumor cells were injected at the yolk sac. Thus we needed to identify a different injection site with which differential metastatic dynamics could be observed at times earlier than 24 hpi. After some tests, we were able to successfully inject tumor cells (~300) into the pericardium^23^ space of the 48 hpf (hour post fertilization) embryos (Fig. 6a) without apparent significant injuries to interfere with the development of the embryos. Extravasation of the melanoma cells at the tail was measured using high resolution confocal microscopy up to 24 hpi (Fig. 6b). Visualization and quantification of the extravasation process demonstrate that TRCs extravasated more efficiently and formed more micrometastases than control melanoma cells at 12 hpi and 24 hpi (Fig. 6c,d); synchronizing the cell cycle time of both TRCs and control cells with 0.1% serum for 24 hrs before injecting them into the fish confirmed those results in Fig. 6 (Supplementary Fig. 8), which was confirmed by measurements using Fluorescence Activated Cell Sorting (FACS) (Supplementary Fig. 9), strengthening the conclusion that it is the more efficient extravasation and not the higher proliferation rates of the TRCs that accounts for more cells at the tail tissue at 12 hpi since the tumor cells were not able to divide at 12 hpi. Treatment of SU5416 (1 μM for 2 hr) to larvae at 20 hpf, a potent inhibitor of new angiogenic and vasculogenic vessel formation in zebrafish^24^, completely blocked the arrival of any tumor cells at the tail at 12 hpi (Supplementary Fig. 10), suggesting that all tumor cells reached the distant site of the tail via vessel circulation and via extravasation from the vessels and not through interstitial spaces. Interestingly, the cells extravasated out of the vessels and through the ECM collectively as a string of cells, i.e., several cells aggregated and clustered together, and not as a single individual cell (Fig. 6c; Supplementary Fig. 11; Movie 1, Movie 2). To further examine to what extent extravasation impacts metastasis, we mixed YFP-TRCs and CFP-Control cells at a 1:1 ratio and co-injected them into the cavity of pericardium. Same numbers of YFP and CFP tumor cells arrived at the tail at 1, 6, and 12 hpi, but more TRCs extravasated than control cells at 12 hpi and at 24 hpi (Supplementary Fig. 12), suggesting that differential targeting in metastasis could be explained largely by efficient extravasation of TRCs alone. Moreover, we found that tumor volumes at the primary injection site and at the secondary sites for TRCs were higher than controls at 48 hpi (Supplementary Fig. 13), indicating that TRCs are grown into bigger metastatic colonies than Cont. These results suggest that TRCs possess greater capacities in generating more micrometastases and bigger metastatic colonies than control cells.

**Figure 7:**
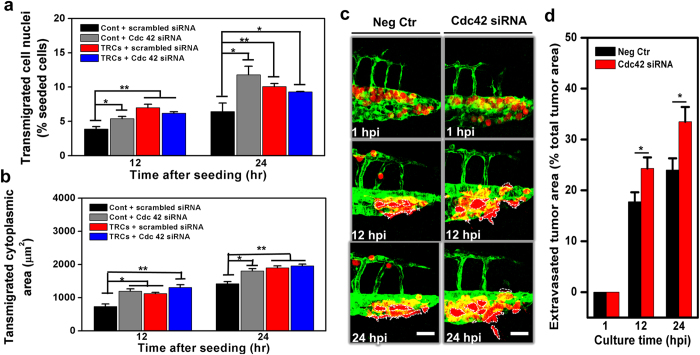
Silencing Cdc42 in control melanoma cells elevates transmigration *in vitro* and extravasation *in vivo*. Transmigration of control melanoma cells or of TRCs through 3-μm pore membrane transwell was measured after the cells were transfected with siRNA to Cdc42 or scrambled siRNA. (**a**) Transmigrated cell nuclei is the percentage of transmigrated cell nuclei (% seeded cells) per view-field. (**b**) Transmigrated cytoplasmic area is the total transmigrated cytoplasmic area per view-field. Mean + s.e.m.; n = 3 independent experiments. *p < 0.05, **p < 0.01, ***p < 0.001. (**c**) Control melanoma cells transfected with scrambled siRNA (Neg Ctr) or Cdc42 siRNA were injected into the pericardium of 48 hpf embryos respectively. Images in each panel show vessel penetration of Neg Ctr (left panels) or Cdc42 siRNA (right panels) at 1, 12, and 24 hpi respectively. Dashed white lines mark the tumor extravasation areas (i.e., various sizes of micrometastases) from vessels to surrounding tissues. Scale bars, 50 μm. (**d**) Quantification of extravasated tumor area relative to the total tumor area at different time points: 1, 12, and 24 hpi. Color code: Zebrafish blood vessels are green, and mouse tumor cells are red. Mean + s.e.m.; n >10 fish per group from 3 independent experiments; *p < 0.05.

Cdc42 is known to facilitate cell spreading^25^, migration^26^, invasion^27^, and transendothelial migration^28^. Based on these published reports, it is logical to predict that downregulation of *Cdc42* would suppress transmigration. Therefore we silenced *Cdc42* with siRNA. Surprisingly, silencing *Cdc42* significantly increased rather than decreased nuclear and cytoplasmic transmigration of control melanoma cells but not TRCs (Fig. 7a,b; Supplementary Fig. 14). Silencing *Cdc42* had no effect on nuclear and cytoplasmic transmigration of TRCs, likely due to the fact that Cdc42 levels in TRCs are already very low^13^. This interpretation is supported by the data that silencing *Cdc42* in control melanoma cells elevated their nuclear and cytoplasmic transmigration to the extent similar to that of TRCs (Fig. 7a,b). Importantly, silencing *Cdc42* in control melanoma cells and injecting these cells into the pericardium led to elevation of extravasated tumor cells in zebrafish, when compared with those transfected with scrambled control, suggesting that extravasation efficiency was increased because of Cdc42 downregulation in those cells (Fig. 7c,d).

**Figure 8:**
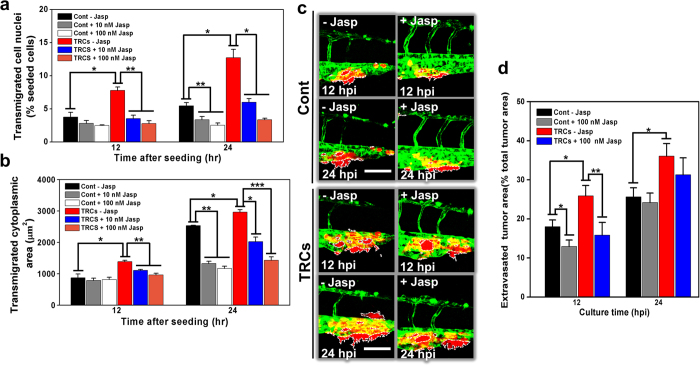
Stiffening TRCs via actin polymerization decreases transmigration *in vitro* and extravasation *in vivo*. Transwell migration of TRCs were compared with that of control melanoma cells before or after actin was polymerized by addition of Jasplakinolide. (**a**) Transmigrated cell nuclei is the percentage of transmigrated cell nuclei (% seeded cells) per view-field. (**b**) Transmigrated cytoplasmic area is the total transmigrated cytoplasmic area per view-field. For (**a**) and (**b**), Mean ± s.e.m.; n = 3 independent experiments. *p < 0.05, **p < 0.01, ***p < 0.001. (**c**) TRCs and Cont pretreated with or without Jasp were injected into the pericardium of 48 hpf embryos respectively. Representative images were acquired with high resolution confocal microscopy (63x objective), showing penetration of Cont ± Jasp (left panels) or TRCs ± Jasp (right panels) at 12 and 24 hpi respectively. Dashed white lines mark the tumor extravasation areas (various sizes of micrometastases) from vessels to surrounding tissues. + Jasp: tumor cells were pretreated with 100 nM Jasp in culture medium for 12 hr before collecting; - Jasp: tumor cells were pretreated with DMSO at the same concentration to Jasp in culture medium for 12 hr before collecting. Scale bars, 100 μm Insets scale bars, 20 μm. (**d**) Quantification of extravasated tumor area relative to the total tumor area at different time points: 12, and 24 hpi. Color code: Zebrafish blood vessels are green, and mouse tumor cells are red. TRCs exhibit higher penetration rates than Cont. Mean ± s.e.m.; n >6 fish per group; 3 independent experiments; *p < 0.05.

To further explore the mechanisms of why extravasation is increased when Cdc42 is downregulated, we examined the impact of Cdc42 on cell rigidity. It is known that Cdc42 facilitates actin polymerization through N-WASP (neuronal Wiskott–Aldrich Syndrome protein) and Arp2/3 (ref. 29) and independently regulates the activation of the JNK(c-Jun N-terminal kinase)-MAPK (mitogen-activated protein kinase) cascade^30^. To test our hypothesis that Cdc42 inhibits cell transmigration *in vitro* and extravasation *in vivo* via F-actin dependent cell rigidity, we increased cell stiffness generically by elevating polymerized actin in the cells with Jasplakinolide^31^ (Jasp) treatment. As expected, in response to the drug that increased F-actin in the cells (Supplementary Figs 15 and 16), there was dose-dependent inhibition of transmigrated cell nuclei and of transmigrated cytoplasmic area (Fig. 8a,b; Supplementary Fig. 17) for both TRCs and control melanoma cells at 24 hrs after seeding, but the inhibition on TRCs was much more dramatic because TRCs were softer than control cells; at 12 hrs after seeding, there was only significant reduction in transmigration for TRCs but not for control melanoma cells. In fact, transmigration of both TRCs and controls was reduced to similar levels at high concentration of Jasp (Fig. 8a,b), suggesting that cell deformability (or softness) was the key determinant of transmigration difference between TRCs and control cells in a 3D matrix.

Based on the data from the transwell experiments, we hypothesize that cell softness is critical for tumor cells to extravasate since penetrating through blood vessels and extracellular matrix (ECM) requires the tumor cells to be very deformable. To test this idea, we treated both control melanoma cells and TRCs with 100 nM Jasp to stiffen the cytoskeleton via actin polymerization for 12 hrs in culture before injecting them into zebrafish. Interestingly, at 12 hpi, Jasp significantly inhibited both Cont and TRCs from extravasation, but the reduction in TRC extravasation was greater (Fig. 8c, d). At 24 hpi, the inhibition by Jasp was no longer significant (Fig. 8d), likely due to resumption of F-actin to the pretreatment level after Jasp was diluted in the fish; this interpretation was supported by the complete disappearance of extra dense F-actin distribution in the cytoplasm of Jasp-treated cells 24 hrs after washout (Supplementary Figs 15 and 16). Taken together, these results suggest that tumor cells, stiffened by high levels of Cdc42 mediated actin polymerization, could limit their penetration out of vessels and through the ECM.

Consistent with what has recently been reported^13^, *Sox2* expression was much higher and *Cdc42* expression was lower in TRCs than in Cont (Supplementary Figs 18 and 19), suggesting that they are inversely associated. To further explore the underlying mechanism for the relationship between the two, we silenced *Sox2* in TRCs (~80% knockdown; Supplementary Fig. 18) and found that *Cdc42* levels increased by ~4-fold (Supplementary Fig. 18 and 19), suggesting that *Sox2*-knockdown induced inhibition of metastasis (see Fig. 4) is likely due to Cdc42 elevation mediated downregulation of cell softness. Interestingly, when *Cdc42* was silenced in Cont (90% knockdown; Supplementary Fig. 18), *Sox2* expression increased by ~4-fold (Supplementary Figs 18 and 19), still much lower than that in TRCs (Supplementary Fig. 18), suggesting that Cdc42 only partially regulates *Sox2* expression, possibly through H3K9 demethylation^13^.

## Discussion

Our current findings reveal that TRCs, a small subpopulation of melanoma cells, exhibit higher proliferation, survival, and metastasis than their counterpart control melanoma cells in a zebrafish model. Specifically, Sox2 expression in the TRCs is critical in extravasation dynamics and formation of micrometastasis. These findings are consistent with previous findings that TRCs are more tumorigenic and metastatic in mice^12^,^13^ but extend those findings to extravasation and micrometastases. Importantly, we find that low Cdc42 and thus high deformability in Sox2-expressing TRCs are the underlying mechanisms of why these cells extravasate better than control cells, opposite from what would be predicted based on earlier findings on the roles of Cdc42 in promoting tumor invasion^27^,^28^. While we find that Sox2 negatively regulates Cdc42, the exact signaling pathway of how Sox2 controls Cdc42 is not clear at this time. Interestingly, even for the invasive subtype of B16 melanoma cells (B16-F10) that are used in our study, we are still able to use soft fibrin gels to mechanically select out a sub-population that has higher extravasation efficiency than the control cells, although these control cells can extravasate pretty well, possibly due to the fact that a small subpopulation within these non-selected control cells are TRCs or possess TRC-like features. In our zebrafish model system, using 2 different injection modes, either into the yolk sac or into the pericardium cavity^23^, reveals similar findings that TRCs are efficient than control melanoma cells in extravasation, suggesting that the observation of high extravasation efficiency by TRCs is not limited to a particular site of the primary tumor. With the injection of tumor cells into the pericardium cavity, we are able to visualize the process of extravasation from 1 hr to 24 hrs, thus avoiding the confounding factor of potential differences in cell proliferation between TRCs and control melanoma cells. While we find that differential extravasation in fish could mostly explain the differences in metastasis between TRCs and control cells, we fully understand that these results do not exclude the possibility that in humans and/or in mice differences in intravasation and rolling may partially explain the difference in metastasis between TRCs and control cells.

Using high resolution confocal microscopy, we find that TRCs penetrate out of the blood vessel more effectively than control melanoma cells. An earlier seminal report shows that the metastatic capability of tumor cells is proportional to the extent of the initial arrest in the capillary^32^, which depends on cell rigidity^33^, since rigid cells are easier to get stuck in the narrow capillaries. In contrast, what we have found, however, is that the soft TRCs extravasate better than their counterpart stiff melanoma cells, suggesting that the ability to squeeze between endothelial cells to get out of blood vessels and through the ECM is a major rate-limiting factor in extravasation and the ensuring micrometastasis formation, although the initial arrest in the vessel is also a necessary step in metastasis. One interesting finding in our study is that extravasating tumor cells appear to invade and move around the secondary sites (e.g., the tail) in aggregates and not as a single cell, consistent with a recent report that circulating tumor cell (CTC) clusters have much higher metastatic potential than single CDCs^34^. The exact mechanism needs to be determined in the future. *In vitro* transmigration assays through 3-μm pore membranes have confirmed the *in vivo* findings that TRCs transmigrate better through these small pores. Previous findings show that the TRCs exhibit similar transmigration rates as the control cells when 8-μm pore membranes are used^12^; together with our current findings, they suggest that matrix pore size is a rate limiting factor for extravasation and tumor cell deformability might be a key determinant of extravasation efficiency, although we fully realize that *in vitro* transwell membranes are a much simpler system (e.g., the membrane pores are stiff and passive materials that cannot be enzymatically modified) than the *in vivo* microenvironment of the fish. A recent paper has shown that *in vivo* tumor tissues have pore sizes of 5-μm or less and that nuclear stiffness conferred by Lamin A is a limiting factor in tumor cell transmigration^35^. The role of Lamin A in extravasation can be explored in the future in the zebrafish model. Recently we have published that TRCs are much softer than the control melanoma cells and this soft behavior persist for a few days in culture^12^,^13^. Our current *in vivo* fish data and *in vitro* transmigration results demonstrate the functional significance of TRC softness measured on flexible substrates in cell culture^12^,^13^. Importantly, when *Cdc42* is silenced in the stiff control melanoma cells, they elevated their transmigration rates to the similar levels as the TRCs, suggesting that high Cdc42 in those cells impede 3D transmigration through narrow pores. These findings are in sharp contrast to a previous finding that Cdc42 promotes cancer cell spreading and transendothelial migration^28^. A major difference is that β1 integrin is downregulated by Cdc42 depletion in the previous study, which leads to significant reduction in cell adhesion and thus transendothelial migration and metastasis. In our study, however, there is no difference in β1 or β3 integrin expression and no difference in cell adhesion between TRCs and control melanoma cells^12^. Importantly, when we treated TRCs with an actin polymerization drug to stiffen the TRCs, their transmigration *in vitro* and extravasation in *vivo* are reduced to the same levels as the control cells, supporting the notion that due to lack of Cdc42, TRCs become soft and deformable, which confers their capacity to more easily penetrate blood vessels and move through narrow spaces in 3D matrices. Jasp is a drug that specifically binds actin and promotes actin polymerization in living cells^36^ and its treatment significantly halts cell locomotion^37^. Our F-actin staining data, transwell invasion data, and *in vivo* fish data all support the role of Jasp in polymerizing actin to decrease cell deformability and thus to slow down extravasation. Since F-actin is an important component for many cell functions, we do not exclude the long-term effect of Jasp on other cell functions such as growth. To further explore if perturbing F-actin dynamics in the opposite direction as Jasp would alter extravasation efficiency, we treated shRNA silenced Sox2 (shSox2) TRCs (that behave like melanoma cell on rigid plastic (Supplementary Fig. 7)) with a low concentration (0.1 μM for 1 hr) of Latrunculin A (LatA), a specific drug that promotes actin depolymerization, to decrease F-actin but not to completely destroy all F-actin (Supplementary Fig. 20). The shSox2 TRCs extravasated more after being treated with LatA than after being treated with dimethyl sulfoxide (DMSO) (Supplementary Fig. 21), suggesting that F-actin (that is inversely proportional to cell deformability) is a key contributor to slowing down extravasation *in vivo*. Together, our findings suggest that it is the cell deformability and not the cell tractions that dictate the extravasation dynamics since it is known that TRCs generate less tractions than control cells^12^, although it is obvious that cells would not be able to invade well if there were no tractions.

Published reports have demonstrated that zebrafish does not start to initiate immune system until ~5 dpf (3 dpi)^20^,^21^,^22^,^23^. In this study we have found that when injected into the yolk sac there is a sudden reduction in the total number of tumor cells starting at 5 dpf, right after detection of the genes encoding T cell receptor subunit in the thymus and elevation of the recombination activation gene 1 (*Rag-1*) at 4 dpf 20,21, consistent with the notion that the removal of these xenografted mouse tumor cells is a result of the immune response, possibly triggered by phosvitin in the yolk sac^38^. Furthermore, our finding that undifferentiated TRCs and their counterpart differentiated melanoma cells^13^ exhibit different extravasation efficiency in early zebrafish embryos suggests that TRCs remain undifferentiated, which is in good accordance with the finding that xenografted melanoma tumor cells from 1 to 4 dpf remain dedifferentiated^22^. To explore the potential differences in extravasation between differentiated TRCs and undifferentiated TRCs in the fish, we differentiated TRCs using a generic soluble chemical retinoic acid (RA) that is known to differentiate these melanoma cells^13^. RA-TRCs transfected with CFP were co-injected with DMSO-TRCs transfected with YFP at 1:1 ratio into the pericardium cavity. RA-TRCs extravasated much less than DMSO-TRCs (Supplementary Fig. 22), suggesting that these undifferentiated TRCs did not differentiate during the extravasation phase (24 hpi) in the fish because if they did, they would extravasate at the same rate as RA-TRCs. We have reported that TRCs can maintain their mechanical memory (plasticity) on hard plastic maximally up to 5 days^13^. To further explore how long TRCs can maintain their phenotypic memory, we re-plated TRCs back to rigid plastic for 7 days. These cells (Rigid-TRCs) were then transfected with pEYFP-N1, and control melanoma cells (Cont) cultured on the rigid plastic were transfected with pECFP-N1. Transfected tumor cells were mixed and co-injected at 1:1 ratio into the cavity of pericardium of 48 hpf embryos. There are no differences in extravasation between Rigid-TRCs and Cont at 12 and 24 hpi (Supplementary Fig. 23), suggesting that returning TRCs to hard plastic for 7 days abolishes their memory and reduces their metastatic potential. One technical issue is whether the injected tumor cells would cause problems for the developing embryos. While we cannot rule out the potential negative impact of injected tumor cells on the normal development of embryos, injecting 200–500 cells per embryo to an embryo of 48 hpf does not appear to have altered the proper embryogenesis to the fish as the fish continues to grow and develop organs and tissues for another 6 to 8 days. Moreover, in a previous report, 100 tumor cells that are injected into an embryo as early as 3.5 hpf can be monitored for another 3 months^22^, suggesting that the burden of the tumor cell number at 48 hpf in our study may not present a significant challenge to the fish development.

Solid tumor metastasis is a complex process that consists of a cascade of events including tumor size expansion at the primary site, local invasion, intravasation, extravasation at secondary sites, formation of micrometastasis, and metastatic colonization^1^. In our current zebrafish model, we have not examined the events of local invasion and intravasation^1^ and the processes of dormancy^10^ and relapse, which are all relevant and important events in cancer. We do find, however, that the injected mouse melanoma TRCs proliferate better than control melanoma cells at the primary site and are more efficient in forming micrometastasis and metastatic colonization at the secondary sites, which is highly dependent on *Sox2* expression, leading to tumor size expansion from 2 dpf to 5 dpf (0 dpi to 3 dpi, Figs 3 and 5), consistent with published results in mice^12^,^13^. Interestingly, we also find that Sox2 is critical in metastatic dynamics at secondary sites, as the number of disseminated tumor foci at various body parts depends on Sox2. This finding extends our previous understanding on the role of Sox2 as a self-renewal gene. Furthermore, we demonstrate that a rate limiting factor for extravasation is the stiffness of the tumor cells that limits their capacity to squeeze between endothelial cells in the blood vessel and through the narrow pores in the ECM.

While it is important to identify specific genes and molecules in tumor metastasis, it is also essential to determine what features of the tumor cells convey their capacity to extavasate into peripheral tissues, as extravasation is a necessary step for formation of micrometastasis and metastatic colonization^1^. It has been shown that a stiffened ECM promotes mammary epithelial cell transformation and cancer progression^39^,^40^,^41^. One might anticipate that the tumor cells in these stiffened ECM should also stiffen and go through the process of epithelial to mesenchymal transition (EMT) to differentiate. However, our current results clearly demonstrate in a zebrafish model that the soft undifferentiated mouse melanoma TRCs extravasate more efficiently than their stiff differentiated counterpart cells, suggesting that stiffened tumor cells may present a rate limiting factor in the extravasation dynamics of the metastasis. Our findings are consistent with previous reports in human tumor cell lines or primary cells that metastatic or invasive cells are generally softer than nonmetastatic or noninvasive cells^42^,^43^,^44^,^45^,^46^. These results are also consistent with a report where cancer cell invasion is decreased when the cells are stiffened via pharmacological activation of myosin II^47^. In addition, our results are in accord with the finding that metastatic tissues are in general very soft^48^ and with the postulate that the ECM presents a physical barrier to cancer progression^49^. To the best of our knowledge, our finding that undifferentiated tumor-repopulating cells extravasate efficiently via the mechanism of high deformability to squeeze between endothelial cells and through the pores of ECM in a zebrafish model (Supplementary Fig. 24) is novel and manipulation of cell deformability (e.g., by stiffening the tumor cells^47^) may present a potential strategy for intervention to slow down or to inhibit extravasation during metastasis.

## Materials and Methods

### Embryos preparation and injection of mouse tumor cells

Breeding zebrafish (Danio rerio), wild-type, Tg(fli1:EGFP), or Tg(kdrl:mCherry), (purchased from Chinese Zebrafish Resource Center, The Institute of hydrobiology, Chinese Academy of Sciences, Wuhan, China), were maintained and embryos were raised under published standard conditions^50^. Zebrafish embryos were kept in 31 °C incubator and the stages (hours or days post-fertilization) in this study were as described^51^. Tumor cell proliferated at this temperature, although more slowly than at 37 °C. At a higher temperature (e.g., 35 °C), fish embryos could not develop normally and many did not survive. All studies using zebrafish were approved by the University’s Animal Care and Use Committee. All the protocols were in strict accordance with guidelines from the Laboratory Animal Training Association and IACUC protocols. 24 h post-fertilization embryos were incubated with egg water containing 0.2 mM 1-phenyl-2-thio-urea (Sigma) to prevent pigmentation. At 48 h post-fertilization, zebrafish embryos were dechorionated with help of a sharp tip forceps and anesthetized with 0.04 mg/ml of tricaine (MS-222, Sigma). Anesthetized embryos were transferred onto a modified agarose gel for microinjection. Tumor cells, resuspended in PBS and calibrated to be ~500 cells (or ~200 cells) with 5~10 nl solution volume, were injected into the yolk sac (or into the pericardium space) of each embryo using an microinjector (ZGP01500, Zgenebio). Non-filamentous borosilicate glass capillaries needles (1.0 mm in diameter, Sutter Instrument) were used for the microinjection after they were pulled into finer diameters of ~20 μm. Diameter of the cell-medium droplet was ~200 μm and each injection duration was ~0.04 sec at 5~20 psi injection pressure. After injection, the fish embryos were immediately transferred to 24-well plate containing egg water and the water was changed daily. After finishing experiments, left-over fish were deeply anesthetized and put on ice to death.

#### Cell lines and culture

Mouse B16-F10 cells were purchased from Wuhan Boster Biology Technology, Ltd. (Wuhan, China); mouse B16-F1 cells and mouse 3T3 cells were from China Center for Type Culture Collection (Wuhan, China). tfRFP-expressing B16-F10 cells were obtained by stably transfecting B16-F10 cells with a plasmid containing the KatushkaS158A gene^17^. B16-F1, tfRFP B16-F10, and 3T3 cells were cultured on rigid dishes with MEM, 1640 and DMEM cell culture medium (HyClone) respectively supplemented with 10% fetal bovine serum (Invitrogen), and 1% penicillin/streptomycin at 37 °C with 5% CO_2_. Cells were passaged every 2–3 days using TrypLE (Invitrogen). TRCs were mechanically selected by being cultured in the 3D soft fibrin gels (90-Pa) for 5 days^12^ from B16 cell lines and both were more metastatic than their respective counterpart control cells. Dexamethasone (Dex), Latrunculin A (Lat A), retinoic acid (RA), dimethyl sulfoxide (DMSO), and ethanol were from Sigma. Angiogenic and vasculogenic blocker SU5416 was purchased from Selleck. Its stock solution was 10 mM in DMSO. The drug was diluted 5000 times such that its final working concentration was 2 μM with 0.02% DMSO in fish medium. After 1 hr incubation with the drug, fresh medium was added to wash out the drug. The drug-treated embryos appeared slightly yellowish, suggesting good absorption.

#### 3D fibrin gel preparation and TRCs collection

Salmon fibrinogen and thrombin were purchased from Reagent Proteins (CA, USA). Three-dimensional fibrin gels were prepared as described previously^12^. In brief, fibrinogen was diluted into 2 mg ml^−1^ with T7 buffer (pH 7.4, 50 mM Tris, 150 mM NaCl). Cells were detached from 2D rigid dishes and cell density was adjusted to 24,000 cells per ml. Fibrinogen and cell solution mixture was made by mixing the same volume of fibrinogen solution and cell solution, resulting in 1 mg/ml fibrinogen and 12,000 cells per ml in the mixture fibrin gels. 250 μl cell and fibrinogen mixture was seeded into each well of 24-well plate and mixed well with pre-added 5 μl thrombin (0.1 Uμl^−1^). The cell culture plate was then incubated in 37 °C cell culture incubator for 10 min. Finally, 1 ml medium containing 10% fetal bovine serum and 1% antibiotics was added. After 5 days, fibrin gel was broken by pipette, then TRCs were collected by centrifuging at 2000 rpm.

#### Fluorescent Plasmids transfection

TRCs were sucked out gently using a pipette, then isolated cells were attached to the top surface of 3D soft fibrin gels (90 Pa) overnight (~10 hrs) before being transfected with fluorescent plasmids. Controls cells were attached to rigid plastics. Adherent TRCs were transfected with pEYFP-N1 and control cells were transfected with pECFP-N1 for 12 hrs following protocols from the company (Youbio, China). In separate experiments, B16-F1 cells cultured on rigid plastic were transfected with pEYFP-N1 and noncancerous mouse 3T3 cells were transfected with pECFP-N1 for 12 hrs. The transfected cells were mixed at 1:1 ratio before being co-injected into fish. Switching the plasmids such that TRCs were transfected with pECFP-N1 and control cells from rigid plastic were transfected with pEYFP-N1 still resulted in more efficient extravasation by TRCs, suggesting that it is the cell behaviors and not the fluorescence that dictate differential extravasation.

#### Fluorescence Activated Cell Sorting (FACS)

For control melanoma cells, tumor cells were seeded onto rigid plastic at ~ 20% density to avoid overcrowding of cells. 12 hrs later, 0.1% FBS containing medium was switched to culture the tumor cells up to 24 hrs to synchronize the cell cycle; for TRCs, cells grew in soft fibrin gels for 4 days, then 0.1% FBS containing medium was changed to culture the TRCs up to 24 hrs to synchronize the cell cycle. TRCs or control melanoma cells that were serum deprived for 0 hr, 12 hrs or 24 hrs were collected, and the cell cycle of all samples was analyzed using flow cytometry.

#### RNA interference

Cells were transfected with shRNA using Lipofectamine 2000 (Invitrogen) according to the manufacturer’s protocol. *Cdc42* siRNAs was acquired from Invitrogen (catalogue number 66023) and the construct sequence is 5′-GGGCAAGAGGAUUAUGACATT-3′ for siRNA 1, and 5′-CCGCUAAGUUAUCCACAGATT-3′ for siRNA 2. *Sox2* shRNAs were obtained from Origene (TG515613). The construct sequence is 5′-GCACTACCAGAGCTAACTCAGATAGTACT-3′ for scrambled shRNA, 5′-AGACGCTCATGAAGAAGGATAAGTACACG-3′ for shRNA 2, and 5′-AGCTACGCGCACATGAACGGCTGGAGCAA-3′ for shRNA 3.

#### Confocal imaging

Anesthetized embryos were imaged in a small drop of Tricaine (0.04 mg/ml) containing water on a glass coverslide. For *in vivo* confocal microscopy, anesthetized fish were housed in a sealed, temperature-controlled chamber (TOKAI Hit, Japan) in 1.2% agarose (low gelling temperature) covered with 0.04 mg/ml Tricaine containing water. We used Leica DMI6000B microscope to image daily and Leica SP8-STED microscope for high resolution images. Excitation was 433 nm for CFP, 488 nm for GFP, 514 nm for YFP, 561 nm for tfRFP and mCherry. For each 3D image, 1- to 5-μm step z-stacks (512 × 512 focal planes) were acquired over a 3~5 min period by using 4X(Leica,N.A.0.10), 10X(Plan Apo, N.A. 0.40), 20X (Plan Apo, N.A. 0.75), or 63X(Plan Apo, N.A. 1.40)objectives. For time series experiments, embryos were kept under anesthesia for up to 24 h.

#### Quantification of tumor size

The tumor size was calculated using the formula of tumor projected area multiply mean fluorescent intensity, which is proportional to the injected tumor number that were counted before injection and after removing the cells from the fish (Supplementary Fig. 1).

#### In vitro transmigration assay

Cell transmigration was assessed using Falcon 24-well inserts (Corning) with 3-μm pores. Before seeding cells, both sides of the membrane were coated with fibrinogen (50 μg/ml) or collagen-I (50 μg/ml) (Sigma) for 2 hr at 37^o^ C. TRCs or control melanoma cells (3 × 10^4^) suspended in 250 μl serum-free MEM (HyClone) were added to the top well of each insert and 750 μl MEM supplemented with 20% FBS was added to the bottom well to establish a gradient across the filter. For drug treatment during transmigration assay, 10 nM or 100 nM Jasplakinolide (Sigma) was added to both the top and the bottom wells. After 12 or 24hr incubation at 37^o^ C with 5% CO_2_, cells were removed from the upper surface of the membranes with a cotton swab, and cells that migrated to the lower surface were fixed with 3.7% formaldehyde (Sigma) for 20 min at room temperature, then stained with 10 μg/ml DiI (for plasma membrane staining) and 10 μg/ml DAPI (for nuclear staining) for 20 min at 37^o^ C. Transmigrated cell coverage was quantified using thresholding in ImageJ software. Transmigrated cell nuclei as percent of seeded cells (Fig. 8a and Supplementary Fig. 7b) were quantified by counting the transmigrated nuclei per view field, divided by the area of the view field ((212 μm × 161 μm), then divided by [seeded cells (30,000)/filter area (0.3 cm^2^)] times 100%, assuming uniform cell seeding at the upper chamber. Transmigrated cytoplasmic area was the total measured area of transmigrated cytoplasm (with Dil to stain for plasma membrane) per view-field. At least 10 randomly chosen view fields from each insert membrane were imaged with Leica DMI6000B microscopy.

#### F-actin staining

Before fixing, all cells were cultured to be attached on the glass bottom dishes coated with 50 μg/ml fibrinogen, the same condition as those used in the transmigration assays. Cells were fixed with 4% paraformaldehyde for 20 min at room temperature and the actin cytoskeleton was stained using 0.76 μM Rhodamine-phalloidin (Sigma) and the DNA was counter-stained with 10 μg ml^-1^ DAPI (Sigma) for 20 min at 37 °C. The samples were rinsed three times with 1X PBS and once in dH_2_O before imaging. F-actin content was quantified along the lines shown using ImageJ.

#### Statistical Analysis

A two-tailed Student’s t-test was used for all statistical analysis.

## Additional Information

**How to cite this article**: Chen, J. *et al.* Efficient extravasation of tumor-repopulating cells depends on cell deformability. *Sci. Rep.* **6**, 19304; doi: 10.1038/srep19304 (2016).

## Supplementary Material

Supplementary Information

Supplementary Movie 1

Supplementary Movie 2
