# Supplementary material for: Efficient extravasation of tumor-repopulating cells depends on cell deformability
Source: Sci Rep. 2016 Jan 20;6:19304. doi: 10.1038/srep19304 (PMC4726408; doi:10.1038/srep19304)
Supplement: Supplementary Information [file srep19304-s1.pdf]

**Supplementary Information**

# Efficient extravasation of tumor-repopulating cells depends on cell deformability

Junjian Chen[1], Wenwen Zhou[1], Qiong Jia[1], Junwei Chen[1], Shuang Zhang[1], Wenting Yao[1], Fuxiang Wei[1], Yuejin Zhang[1], Fang Yang[1], Wei Huang[1], Yao Zhang[1], Huafeng Zhang[2], Yi Zhang[2], Bo Huang[2,3], Zhihong Zhang[4], Haibo Jia[1,*], and Ning Wang[1,5,*]

[1]Laboratory for Cellular Biomechanics and Regenerative Medicine, Department of Biomechanical Engineering, School of Life Sciences, Huazhong University of Science and Technology, Wuhan, Hubei 430074, China

[2]Department of Biochemistry and Molecular Biology, Tongji Medical College, Huazhong University of Science and Technology, Wuhan, Hubei 430030 China

[3]Department of Immunology, Institute of Basic Medical Sciences of Chinese Academy of Medical Sciences, Beijing 100005 China

[4]Britton Chance Center for Biomedical Photonics, Wuhan National Laboratory for Optoelectronics-Huazhong University of Science and Technology, Wuhan, Hubei 430074, China

[5]Department of Mechanical Science and Engineering, College of Engineering, University of Illinois at Urbana-Champaign, Urbana, IL 61801 USA

Key words: cancer metastasis, extravasation, tumor cell softness, micrometastasis, metastatic colonization

*Send correspondence to:

Dr. Ning Wang, nwangrw@illinois.edu

Dr. Haibo Jia, haibo.jia@mail.hust.edu.cn

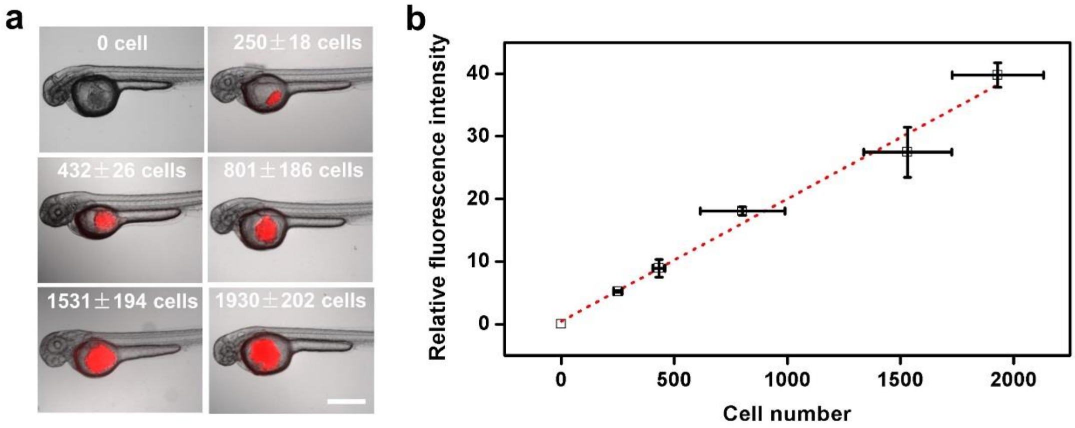


**Supplementary Fig. 1. Calibration of cell number in zebrafish.** (**a**) Representative images of 2 dpf zebrafish injected with different numbers of tumor cells into the yolk. Red color are tfRFP B16 melanoma cells. (**b**) Calibration of the number of injected tumor cells into the yolk with relative fluorescence intensity (corresponding to the projected fluorescence area multiplying fluorescence intensity). Injected cells were counted using a cell counter *in vitro* before and after injection. Projected fluorescence area and intensity were quantified using ImageJ. Dashed line is the linear fit of the data. Mean±s.e.m.; n≥10 larvae. Scale bar, 500 μm.

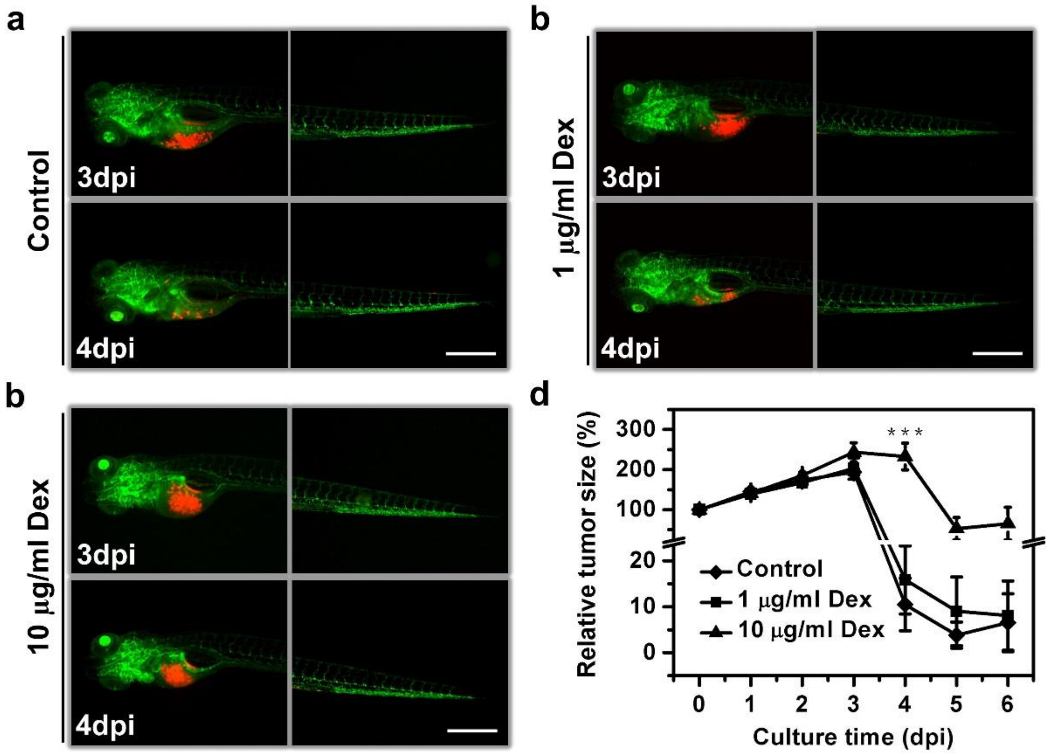


**Supplementary Fig. 2. Immune suppression facilitates tumor cell survival in the yolk.** Approximately 500 tfRFP-B16 cells were injected into the yolk sac of fish at 2 dpf. At 4 dpf (2 dpi), dexamethasone (**Dex**) dissolved in ethanol was added into the water at a final concentration of 1 or 10 μg/ml to fish culture for 2 days to inhibit the immune system. The corresponding ethanol-containing water (0.1%) was used as a negative control (**Control**). Vessels are green and tumor cells are red. Scale bars, 500 μm. (d) Quantification shows that tumor cells survived much better in fish when immune system was inhibited by 10 μg/ml **Dex**. Mean±s.e.m.; n=12 larvae for each condition; ***$p < 0.001$.

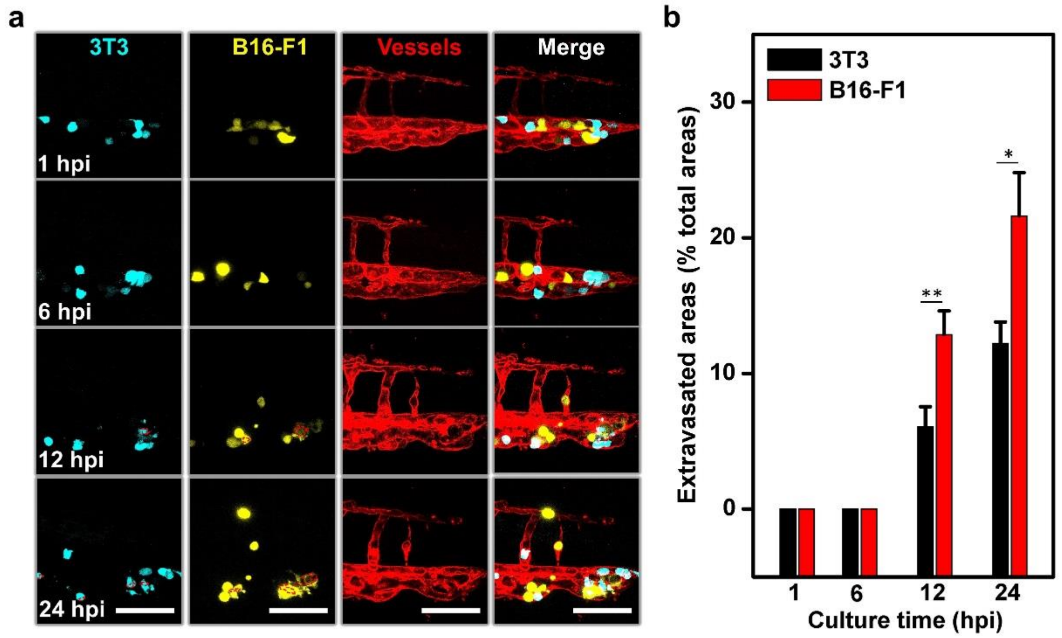


**Supplementary Fig. 3. Noncancerous cells extravasate much less than melanoma cells *in vivo*.** (**a**) Mouse B16-F1 melanoma cells were transfected with pEYFP-N1 and non-cancerous mouse 3T3 cells were transfected with pECFP-N1 respectively, then mixed and co-injected at 1:1 ratio (total cell number ~500) into the cavity of pericardium of 48 hpf embryos. Images in the first two panels show penetration area of normal (left panels) and cancerous (right panels) cells at 1, 6, 12, and 24 hpi respectively, and the next two panels show images of vessels and 3-color overlays. Dashed red lines mark cell extravasation areas from vessels to surrounding tissues. Scale bars, 100 μm. (**b**) Quantification of extravasated area relative to the total area at the fish tail at different time points: 1, 6, 12, and 24 hpi. Color code: Zebrafish blood vessels are red, 3T3 cells are cyan, and B16-F1 cells are yellow. Mean$\pm$s.e.m.; n = 28 embryos; *p< 0.05; ** p < 0.01.

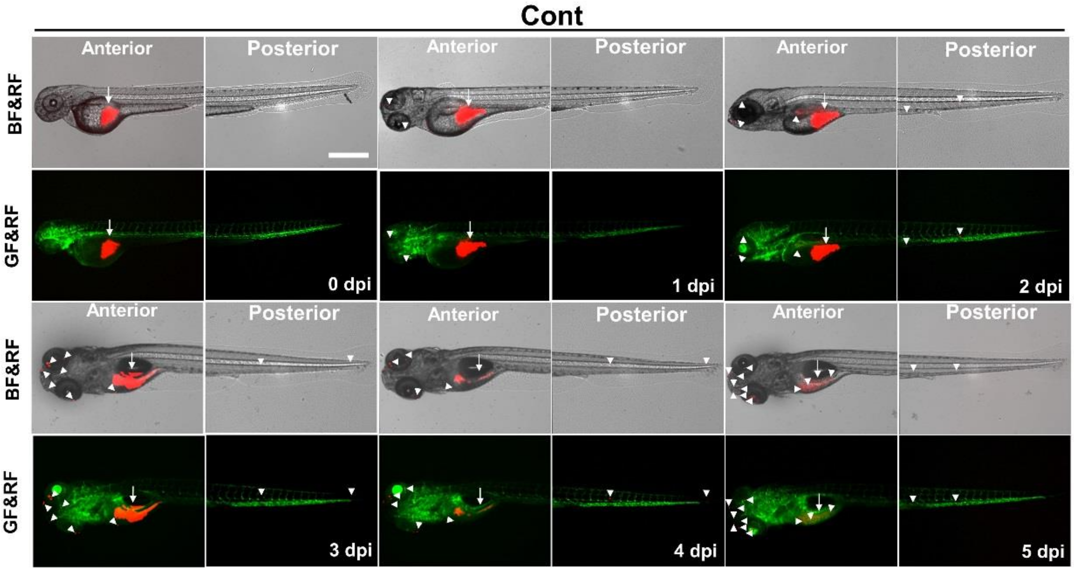


**Supplementary Fig. 4. Time-course analysis of metastasis of control melanoma cells.** Approximately 500 melanoma cells cultured on rigid dish were injected into the yolk sac of zebrafish and the tumor progression were monitored every 24 hr. Representative images from a fish were acquired from 0 dpi to 5 dpi. Arrows indicate primary tumors, arrowheads indicate disseminated tumor foci or tumor foci aggregates. Color code: zebrafish blood vessels are green, and melanoma cells are red. Scale bars, 500 μm.

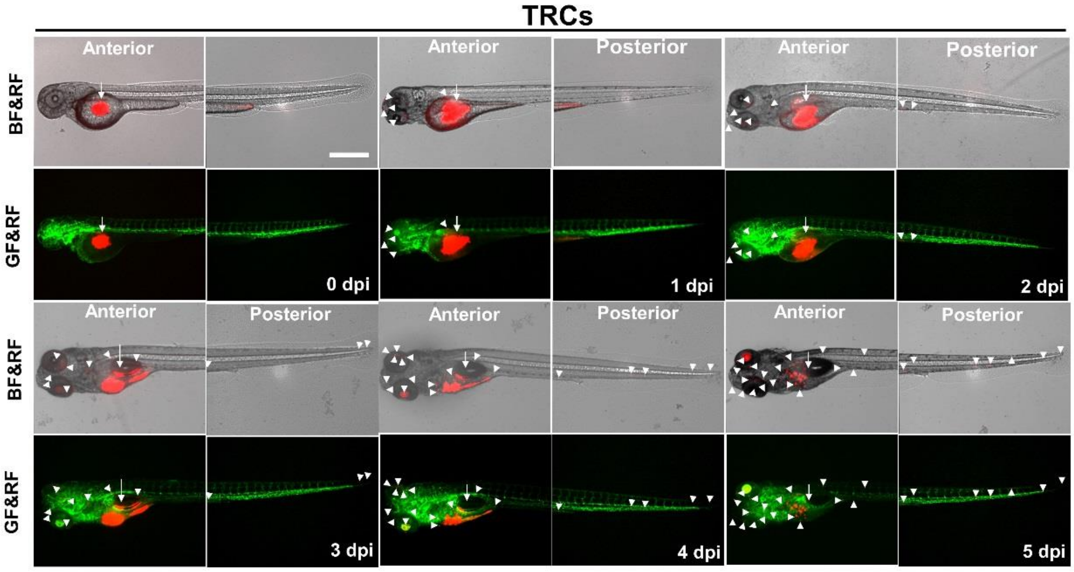


**Supplementary Fig. 5. Time-course analysis of metastasis of TRCs.** The protocols and labels were identical to those in Supplementary Fig. 2 except that TRCs were injected. Scale bars, 500 μm.

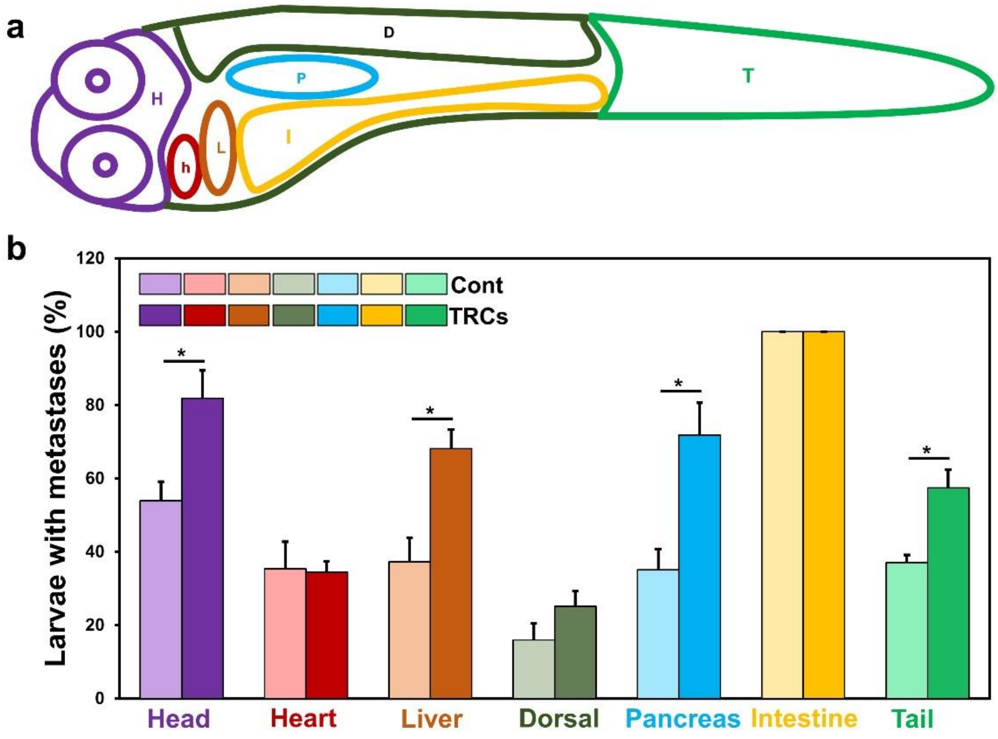


**Supplementary Fig. 6. TRCs injected zebrafish larvae have higher metastases.** (**a**) Schematic of dividing the fish body into different parts: H=Head; h=Heart; L=Liver; P=Pancreas; I=Intestine; D=Dorsal; T=Tail. (**b**) Percentage of larvae exhibiting metastases in various body parts. Note that while there are no differences between TRCs and Cont at Heart, Dorsal, and Intestine, there are significant differences at Head, Liver, Pancreas, and Tail. Mean±s.e.m.; n= 59 larvae for Cont and 22 larvae for TRC from 3 independent experiments. *, $p<0.05$.

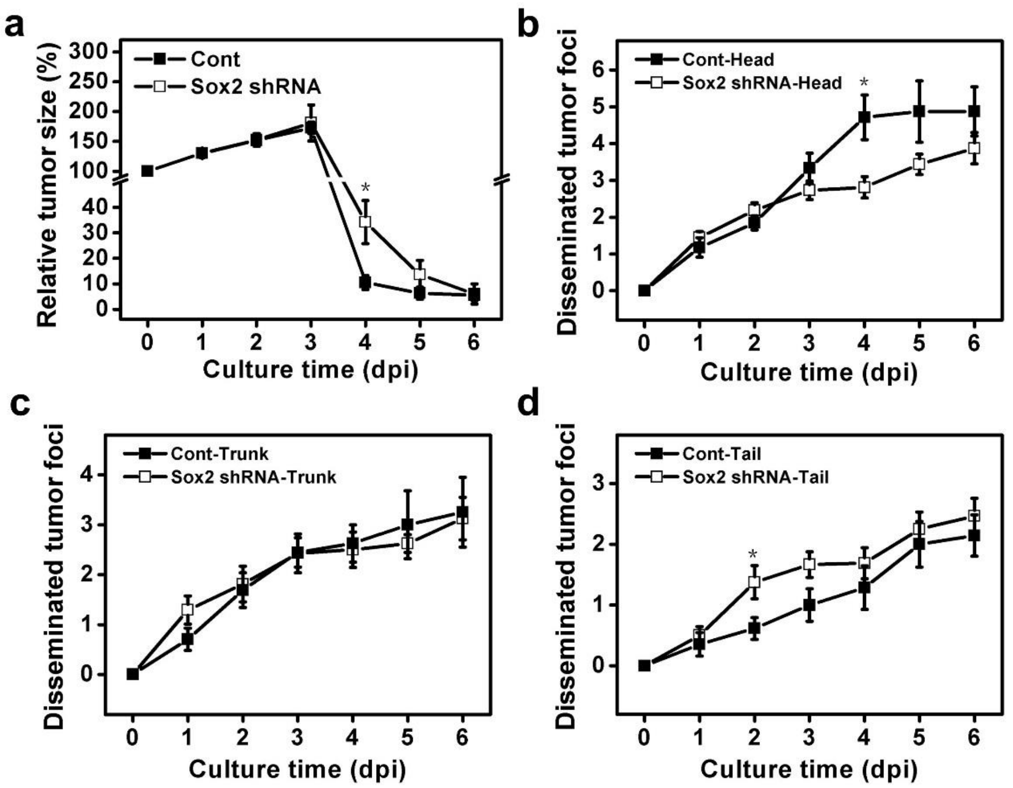


**Supplementary Fig. 7. TRCs treated with shRNA Sox2 behave quantitatively similar to the cells cultured on rigid plastic.** Parts of data in (**a**), (**b**), (**c**), and (**d**) of Fig. 3 (**Cont**) and of Fig. 5 (**Sox2 shRNA**) were re-plotted as (**a**), (**b**), (**c**), and (**d**) in this figure. **Cont**: cell cultured on rigid plastic. **Sox2 shRNA**: TRCs treated with shRNA for Sox2. The data were quantitatively similar except few points, suggesting that silencing Sox2 in TRCs makes them behave like the cells cultured on rigid plastic. Mean±s.e.m; all numbers were the same as those in Fig. 3 and Fig.5; *, $p<0.05$.

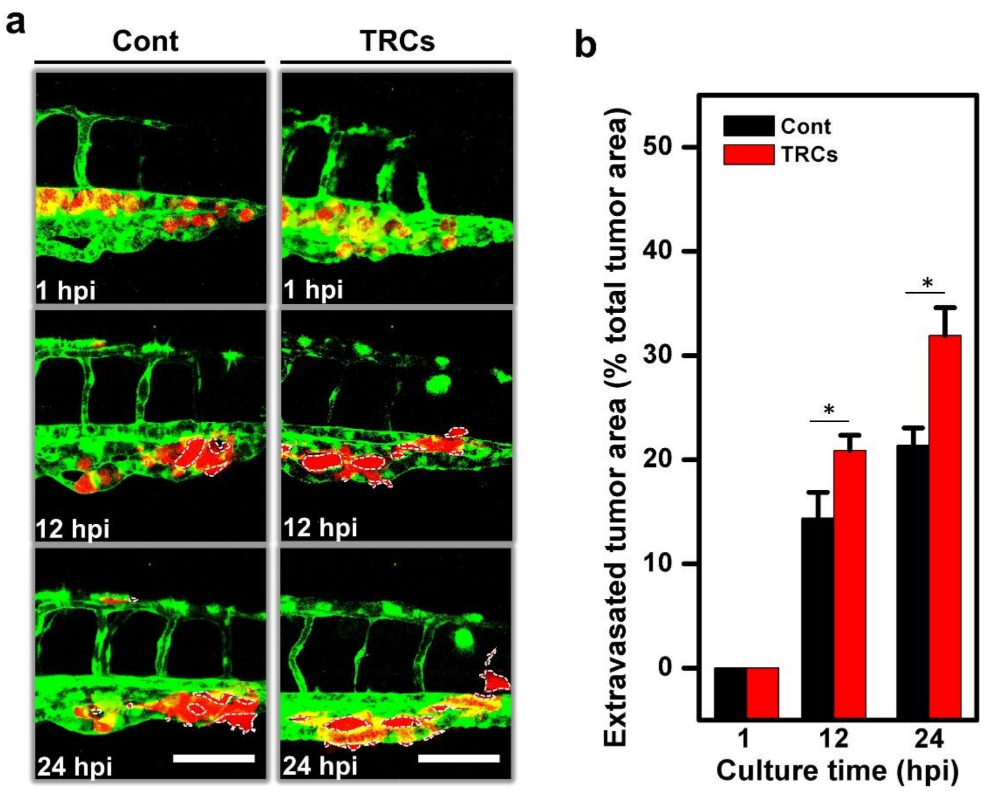


**Supplementary Fig. 8. Serum deprivation demonstrates that TRCs extravasate more effectively than control melanoma cells *in vivo*.** (**a**) TRCs and Cont after serum deprivation (0.1% serum containing medium cultured tumor cells for 24 hrs before injection) were injected into the pericardium of 48 hpf embryos respectively. Images in each panel show vessel penetration of Cont (left panels) or TRCs (right panels) at 1, 12, and 24 hpi respectively. Dashed white lines mark the tumor extravasation areas (i.e., various sizes of micrometastases) from vessels to surrounding tissues. Scale bars, 100 μm. (**b**) Quantification of extravasated tumor area relative to the total tumor area at different time points: 1, 12, and 24 hpi. Color code: Zebrafish blood vessels are green, and mouse tumor cells are red. TRCs exhibit higher penetration rates than Cont. Mean$\pm$s.e.m.; n>6 fish per group; $\geq$3 independent experiments. *$p < 0.05$.

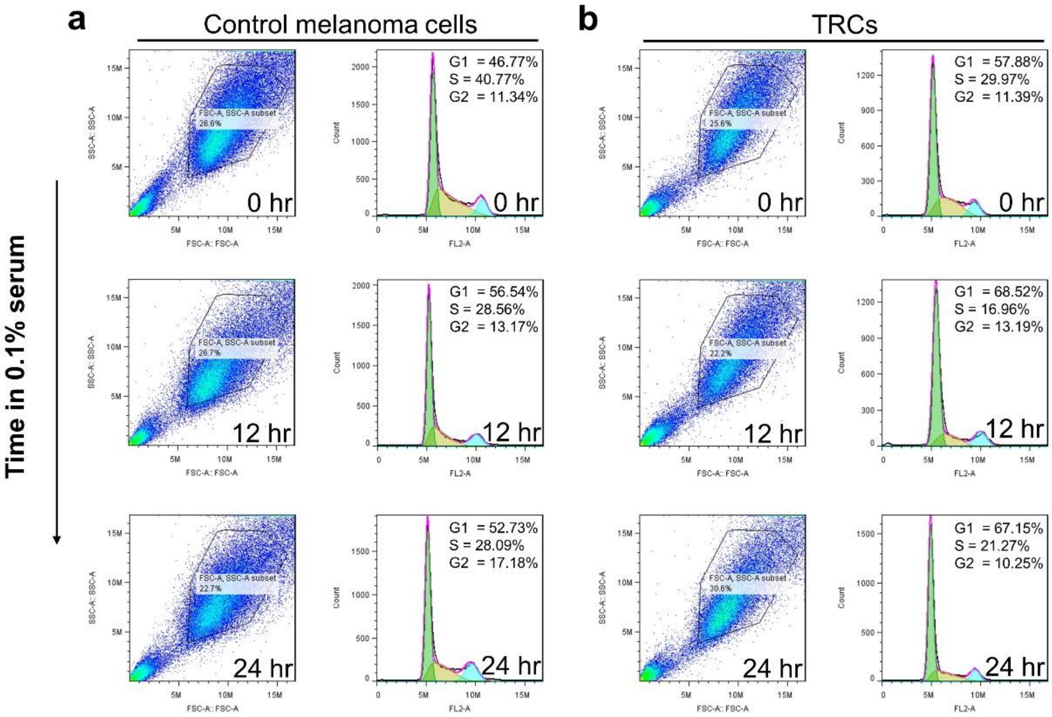


**Supplementary Fig. 9. Serum deprivation increases control melanoma cells and TRCs in G1 phase. (a**) Analysis of the cell cycle of control melanoma cells after serum deprivation (0.1% serum) for 0 hr, 12 hrs, and 24 hrs. Images in the left panel show cell subsets gated for cell cycle analysis. Images in the right panel show cell cycle analysis. (**b**) Analysis of the cell cycle of TRCs after serum deprivation (0.1% serum) for 0 hr, 12 hrs, and 24 hrs. Images in the left panel show cell subsets gated for cell cycle analysis. Images in the right panel show cell cycle analysis.

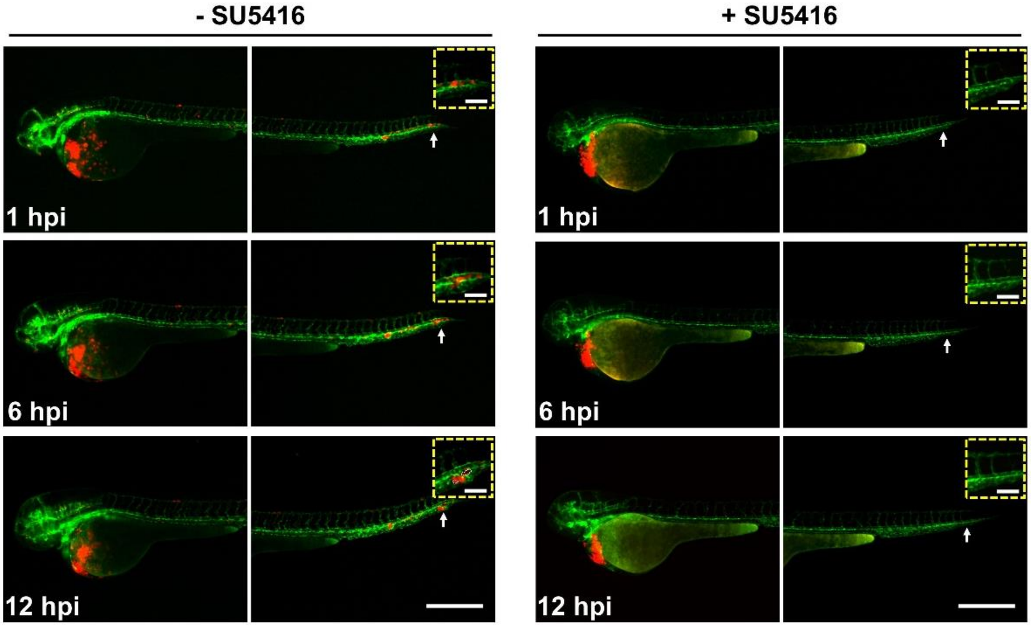


**Supplementary Fig. 10. Injected tumor cells move to the tail via blood vessels.** Tg (flil1:egfp) embryos at 20 hpf were treated with 2 μM SU5416 for 1 hr (+**SU5416**) to inhibit vasculogenesis[24]; the control fish were treated with 0.02% DMSO for 1 hr (-**SU5416**). After 1 hr, SU5416 or DMSO was washed out by changing fish media. At 48 hpf, tfRFP-B16 cells were injected into the pericardium cavity of fish. Representative images show that tumor cells moved to the tail in a drug-free larva, while no tumor cells moved to the tail in a drug-treated larva after new vessels were inhibited by SU5416. Insets are enlarged images from each corresponding tip of the tail indicated by white arrows. Dashed white lines mark extravasated tumor cells at 12 hpi. Vessels are green and tumor cells are red. –**SU5416**, 6 other larvae exhibit similar behaviors; +**SU5416**, 3 other larvae exhibit similar behaviors. Scale bars, 500 μm. Insets, 100 μm.

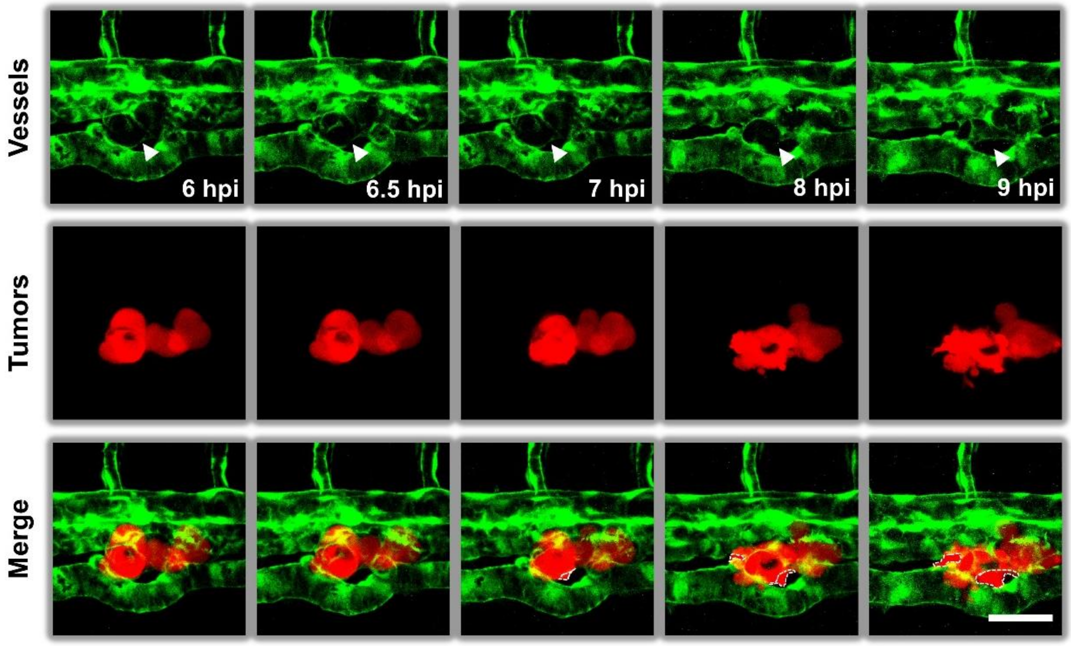


**Supplementary Fig. 11. High resolution microscopy of time-course of TRCs extravasation.** TRCs arrested at the blood vessels of the zebrafish tail extravasated gradually into peripheral tissues. Time-lapse images were acquired using confocal microscopy at 30-min intervals. White arrowheads indicated the boundaries of vessels. White dashed lines circled projected areas of extravasated TRCs. Color code: zebrafish blood vessels are green, and melanoma cells are red. Scale bar: 50 μm.

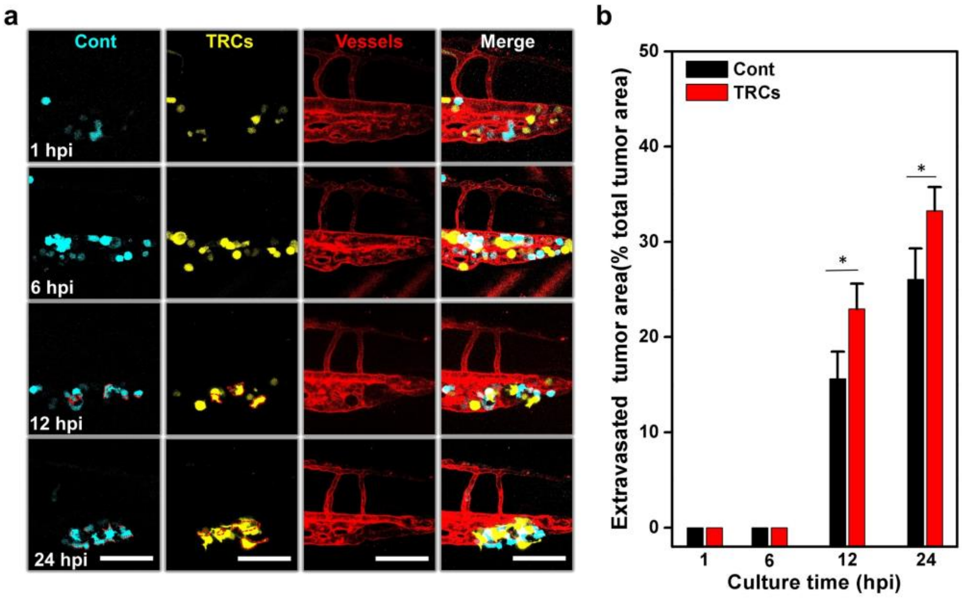


**Supplementary Fig. 12. TRCs extravasate more effectively than control melanoma cells *in vivo* after co-injection.** (**a**) TRCs were transfected with pEYFP-N1 and Cont were transfected with pECFP-N1 respectively, then mixed and co-injected at 1:1 ratio into the cavity of pericardium of 48 hpf embryos. Images in the first two panels show penetration area of Cont (left panels) or TRCs (right panels) at 1, 6, 12, and 24 hpi respectively, and the next two panels show images of vessels and 3-color overlays respectively. Dashed red lines mark the tumor extravasation areas from vessels to surrounding tissues. Scale bars, 100 µm. Total tumor cell volumes were quantitatively similar at 1, 6, and 12 hpi for Cont and TRCs, suggesting that the cell numbers were similar. (**b**) Quantification of extravasated tumor area relative to the total tumor area at different time points: 1, 6, 12, and 24 hpi. Color code: Zebrafish blood vessels are red, Cont are cyan, and TRCs are yellow. Mean±s.e.m.; n>6 fish per group; ≥3 independent experiments. *$p < 0.05$.

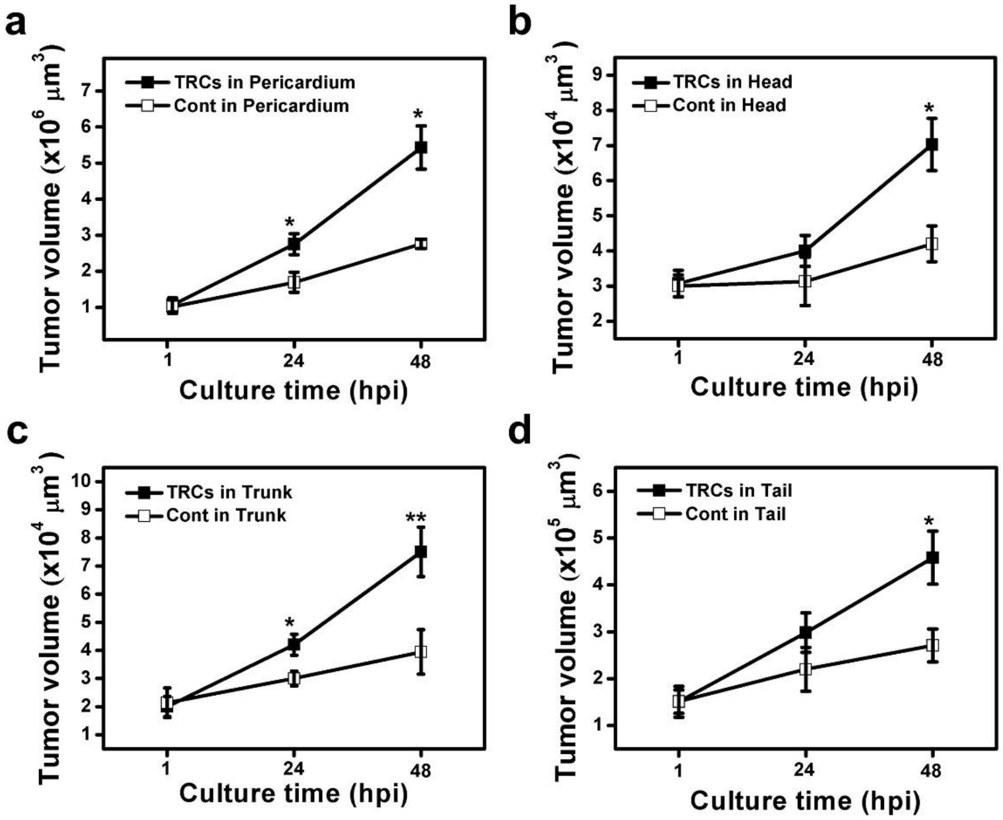


**Supplementary Fig. 13. Quantification of tumor volume at primary and secondary sites.** Tumor cells were injected at the pericardium space of embryos at 48 hpf. (**a**) Quantification of tumor volumes at the primary site. (**b-d**) Quantification of tumor volumes at the secondary site of the head, the trunk, or the tail. All data showed that TRCs grew bigger metastatic colonies than Cont at 48 hpi. Mean$\pm$s.e.m.; n=3 separate experiments; *$p<0.05$; **$p<0.01$.

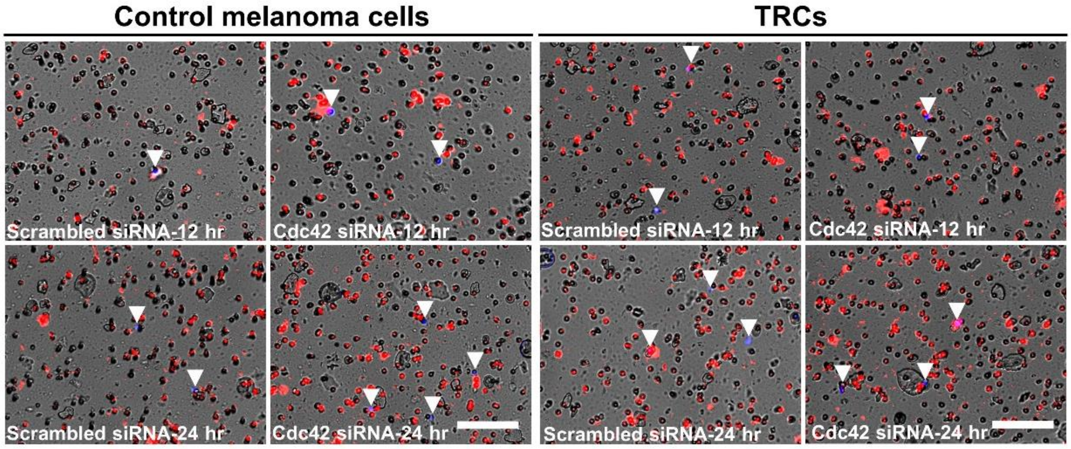


**Supplementary Fig. 14. Silencing Cdc42 increases transmigration of control melanoma cells.** Membranes of 3-μm pores were pre-coated with fibrinogen (50 μg/ml). Representative images are shown to compare Cont transfected with Cdc42 siRNA to that transfected with scrambled siRNA, and TRCs transfected with Cdc42 siRNA to that transfected with scrambled siRNA. Scale bars: 50 μm.

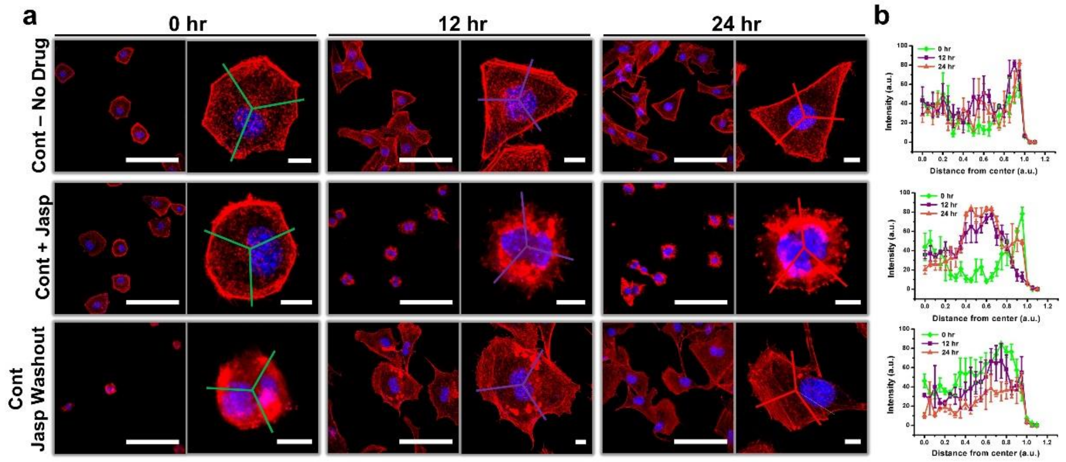


**Supplementary Fig. 15. Jasplakinolide polymerizes actin in control melanoma cells.** (**a**) Representative images of control melanoma cells without (top) or with (middle) Jasplakinolide (100 nM) or Jasp washout (bottom) for different durations (0, 12, or 24 hrs). The cells were plated onto fibrinogen (50 μg/ml)-coated glass-bottomed dishes for 2 hrs for all "0 hr" conditions. Attached tumor cells were fixed and stained for F-actin with Rhodamine-phalloidin (red) and for nucleus with DAPI (blue) respectively. **Cont – No Drug**: no drug treatment. **Cont + Jasp**: treated with Jasplakinolide after cell attachment for 2 hr. This condition simulates that of transwell assays during drug treatment. **Cont Jasp Washout**: the cells were pretreated with 100 nM Jasplakinolide for 12 hrs before attachment. The drug was washed out after cell attachment. This condition simulates that of the tumor cells that are pre-treated with the drug and then injected into the zebrafish. Left image of each panel: low magnification; scale bars: 100 μm. Right image of each panel: high magnification; scale bars, 10 μm. Lines in each panel, green, blue, and red were used to acquire the corresponding fluorescence intensity data shown in (b). (**b**) Quantification of F-actin distribution along the center of the cell (denoted as 0.0) and at the cell edge (denoted as 1.0). a.u.=arbitrary unit. Note that some extra-F-actin polymerization still remains after Jasplakinolide is washed out for 12 hr but it completely disappears 24 hr after washout. Mean ± s.e.m.; n=3 lines from the representative cell, randomly chosen from cells in the low magnification field of view. Another independent experiment showed similar results.

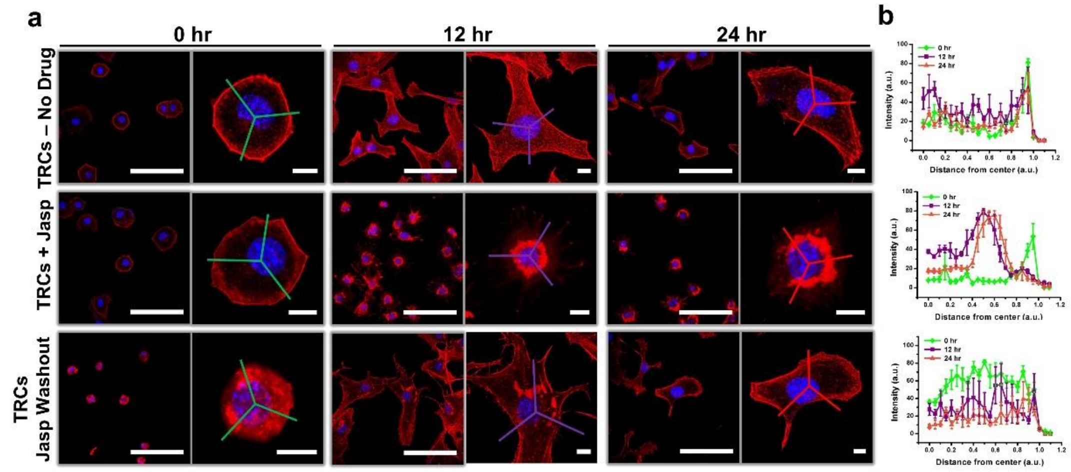


**Supplementary Fig. 16. Jasplakinolide polymerizes actin in TRCs.** (**a**) Representative images of TRCs without (top) or with (middle) Jasplakinolide (100 nM) or Jasp washout (bottom) for different durations (0, 12, or 24 hrs). The cells were plated onto fibrinogen (50 μg/ml)-coated glass-bottomed dishes for 2 hrs for all "0 hr" conditions. Attached tumor cells were fixed and stained for F-actin with Rhodamine-phalloidin (red) and for nucleus with DAPI (blue) respectively. **TRCs – No Drug**: no drug treatment. Note that at 12 and 24 hr, the F-actin accumulates mostly around the nucleus, whereas the rest of the cytoplasm is very dim (very low F-actin). **TRCs + Jasp**: treated with Jasplakinolide after attachment for 2 hr. This condition simulates that of transwell assays during drug treatment. **TRCs Jasp Washout**: the cells were pretreated with 100 nM Jasplakinolide for 12 hrs before attachment. The drug was washed out after cell attachment. This condition simulates that of the tumor cells that are pre-treated with the drug and then injected into the zebrafish. Left image of each panel: low magnification; scale bars: 100 μm. Right image of each panel: high magnification; scale bars, 10 μm. Lines in each panel, green, blue, and red were used to acquire the corresponding fluorescence intensity data shown in (b). (**b**) Quantification of F-actin distribution along the center of the cell (denoted as 0.0) and at the cell edge (denoted as 1.0). a.u.=arbitrary unit. Note that some extra-F-actin polymerization still remains after Jasplakinolide is washed out for 12 hr but it completely disappears at 24 hr after washout. Mean ± s.e.m.; n = 3 lines from the representative cell, randomly chosen from cells in the low magnification field of view. Another independent experiment showed similar results.

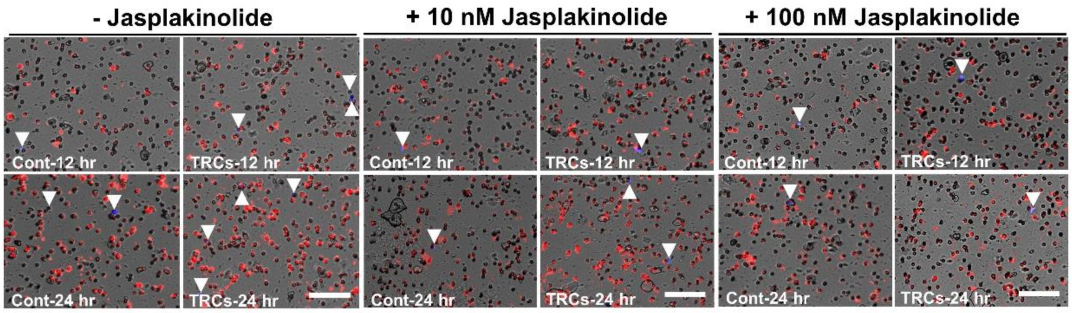


**Supplementary Fig. 17. Actin polymerization downregulates transmigration rates.** Membranes of 3-μm pores were pre-coated with fibrinogen. Representative images are shown to compare TRCs with Cont at three conditions: without drug (left panel), +10 nM Jasplakinolide (middle panel), and +100 nM Jasplakinolide (right panel). Scale bars: 50 μm.

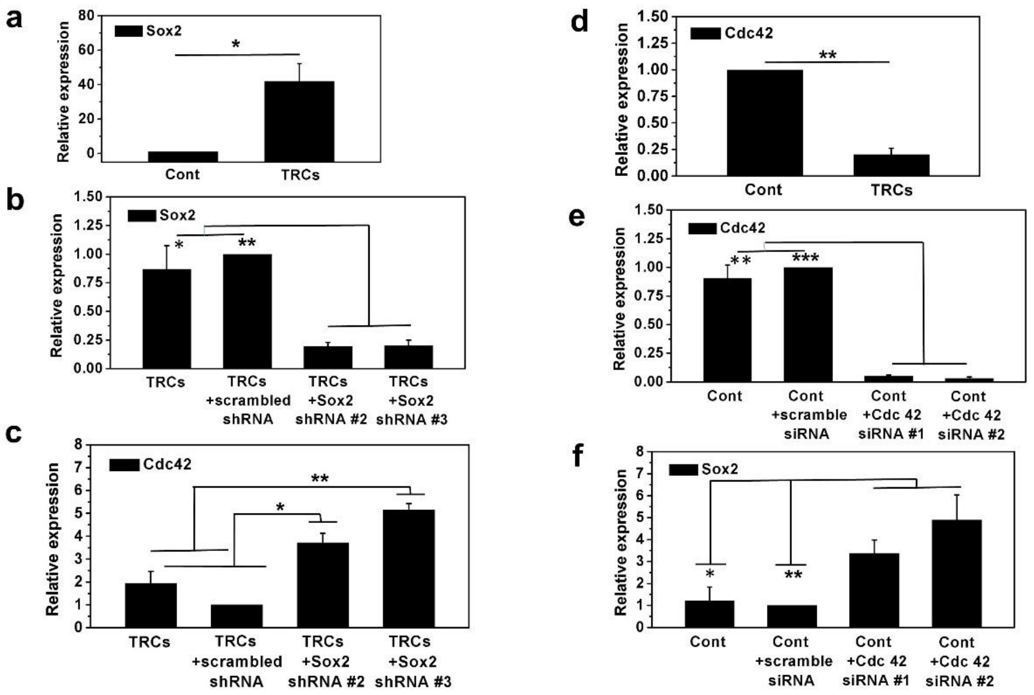


**Supplementary Fig. 18. *Sox2* negatively regulates *Cdc42* in TRCs.** Real time qPCR of *Cdc42* or *Sox2* was measured after *Sox2* or *Cdc42* was silenced with shRNA or siRNA. (**a**) TRCs express much higher levels of *Sox2* than control melanoma cells (Cont). (**b**) *Sox2* is knocked down by ~80% with 2 different shRNAs. (**c**) Silencing *Sox2* led to ~4-fold increase in *Cdc42* expression in TRCs. (**d**) TRCs express lower level of Cdc42 compared with control melanoma cells. (**e**) *Cdc42* is knocked down by >90% with 2 different siRNAs in control melanoma cells (Cont). (**f**). Silencing *Cdc42* led to ~4-fold increase in *Sox2* expression in Cont. Mean ± s.e.m; n = 3 independent experiments; * $p < 0.05$, ** $p < 0.01$, *** $p < 0.001$.

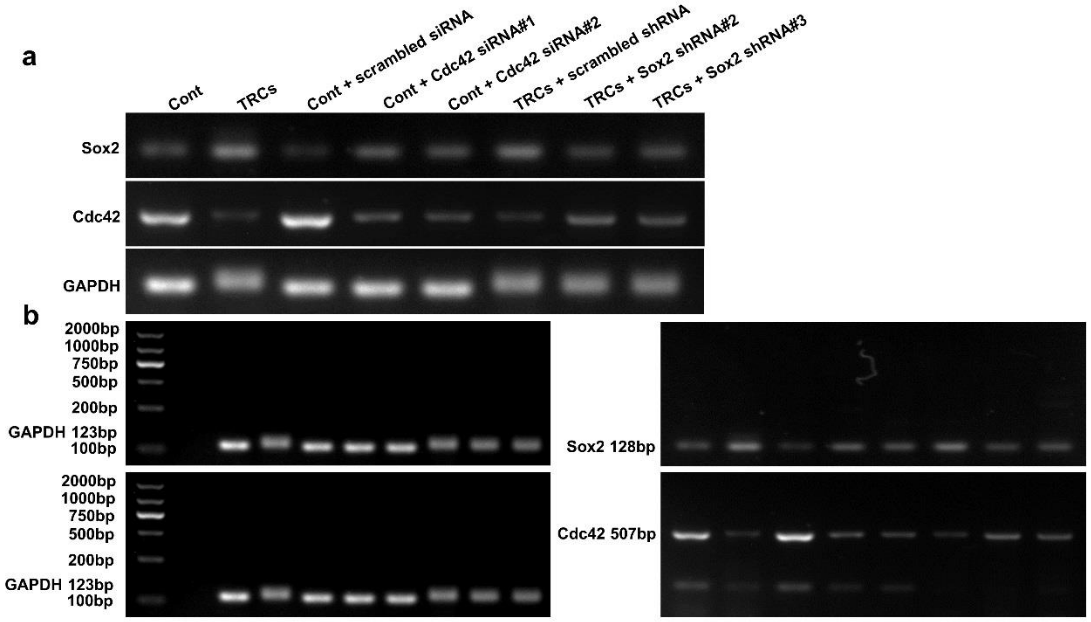


**Supplementary Fig. 19. *Sox2* and *Cdc42* are inversely proportional.** (**a**) *Sox2* and *Cdc42* mRNA expressions were measured with RT-PCR. **Cont**: B16-F1 cells cultured on rigid plastic. **TRCs**: melanoma cells were cultured in 90-Pa fibrin gels for 5 days and without transfection. **Cont + scrambled siRNA**: Cont were transfected with Ccd42 scrambled siRNA. **Cont + Cdc42 siRNA #1**: Cont were transfected with Ccd42 siRNA #1. **Cont + Cdc42 siRNA #2**: Cont were transfected with Ccd42 siRNA #2. **TRCs + scrambled shRNA**: TRCs were transfected with Sox2 scrambled shRNA. **TRCs + Sox2 shRNA #2**: TRCs were transfected with Sox2 shRNA #2. **TRCs + Sox2 shRNA #3**: TRCs were transfected with Sox2 shRNA #3. Note that knocking down *Cdc42* in Cont increases *Sox2* expression, while inhibiting *Sox2* in TRCs promotes *Cdc42* expression. Images are representatives of 2 independent RT-PCR experiments. (**b**) Original images with calibration markers show the gene sizes of *GAPDH, Sox2,* and *Cdc42* in (a).

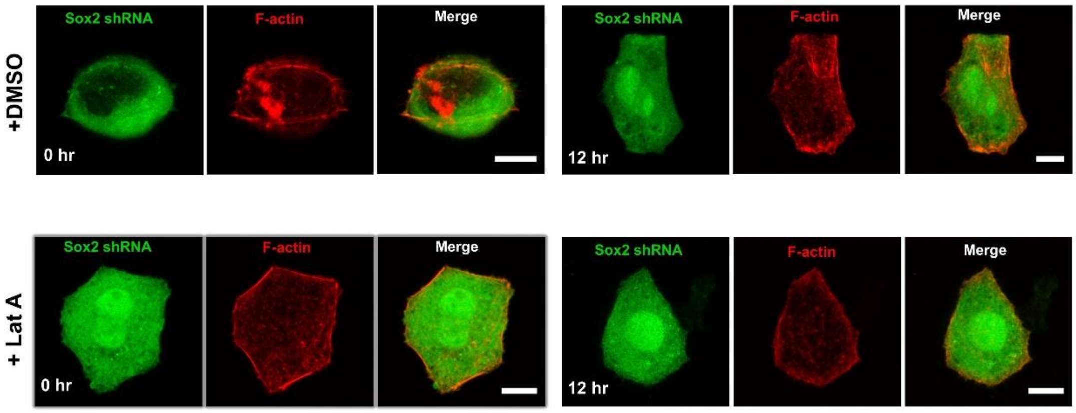


**Supplementary Fig. 20. Latrunculin A depolymerizes actin in shSox2 TRCs.** TRCs were transfected with Sox2-shRNA (shSox2) on top of soft fibrin gels for 12 hrs. shSox2 TRCs were then treated with either 0.1 μM Latrunculin A (Lat A) or 0.004%DMSO (a dissolvent for Lat A) for 1 hr. Treated cells were collected and cultured in glass-bottomed dish (pre-coated with 50 μg/ml fibrinogen) at 37 ℃ for 2 hrs (set as "0 hr") and 12 hrs in Lat A-free medium. Attached cells at different culturing time points were fixed with 4% formaldehyde and stained with Rhodamine–phalloidin. **+DMSO:** representative images show 0.004% DMSO-treated shSox2 TRCs (green), F-actin (Red), and merge at 0 or 12 hrs. **+ Lat A:** representative images show 0.1 μM Lat A-treated shSox2 TRCs at 0 or 12 hrs. Scale bars, 10 μm. Note that numerous actin bundles are in the cytoplasm of **+DMSO** cells but few actin bundles in the cytoplasm of **+Lat A** cells except at the cell periphery, suggesting that Lat A-treated cells are more deformable.

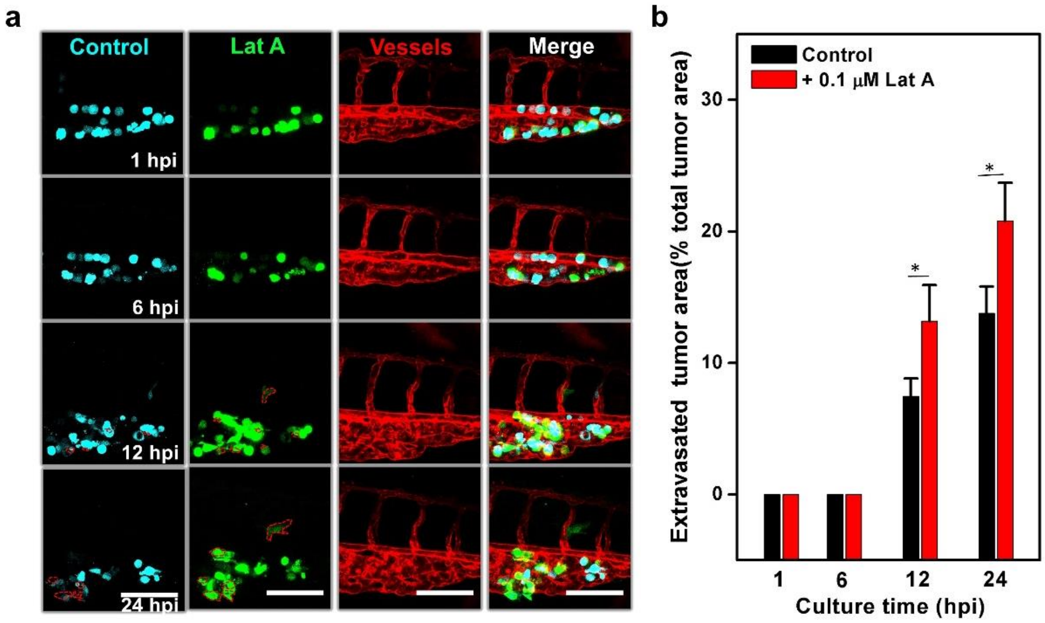


**Supplementary Fig. 21. Softening shSox2 TRCs via actin depolymerization increases tumor extravasation in fish.** TRCs were transfected with GFP-Sox2 shRNA (green) on the top of soft fibrin gels for 12 hrs, then treated with 0.1 μM Latrunculin A (Lat A) for 1 hr and 0.004% DMSO was used as a control. For DMSO-treated group, pECFP-N1 plasmids were co-transfected into GFP-shSox2-TRCs; for Lat A-treated group, non-fluorescent vector plasmids were co-transfected into GFP-shSox2-TRCs. Two types of tumor cells were mixed and co-injected at 1:1 ratio into the cavity of pericardium of 48 hpf embryos. (**a**) Representative images show tumor cells co-extravasation at 1, 6, 12, and 24 hpi. Dashed red lines mark tumor extravasation areas from vessels to surrounding tissues. DMSO-treating cells (control) are cyan, Lat A-treating cells (Lat A) are green, and vessels are red. Scale bars, 100 μm. (**b**) Quantification of extravasated tumor areas at 1, 6, 12, and 24 hpi. Note that 0.1 μM Lat A significantly increased the extravasation of shSox2-TRCs. Mean±s.e.m.; n=18 larvae; *$p < 0.05$.

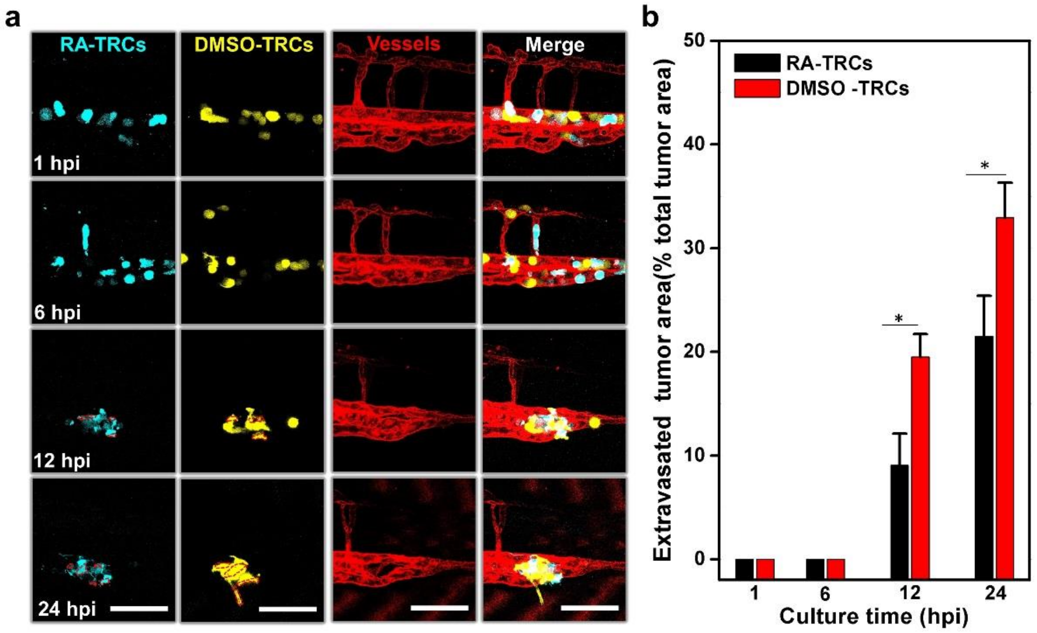


**Supplementary Fig. 22. Differentiation of TRCs with retinoic acid inhibits extravasation of TRCs *in vivo.*** (**a**) TRCs treated with retinoic acid (RA, 10 µM) or DMSO (0.1%) for 12 hrs were transfected with pECFP-N1 and pEYFP-N1 respectively. The cells were mixed and co-injected at 1:1 ratio into the pericardium of 48 hpf embryos. Images in the first two panels show penetration area of RA-TRCs (left panels) or DMSO-TRCs (right panels) at 1, 6, 12, and 24 hpi respectively, and the next two panels show zebrafish vessels and merges respectively. Dashed red lines mark the tumor extravasation areas (i.e., various sizes of micrometastases) from vessels to surrounding tissues. Scale bars, 100 µm. (**b**) Quantification of extravasated tumor area relative to the total tumor area at different time points: 1, 6, 12, and 24 hpi. Color code: Zebrafish blood vessels are red, RA-TRCs are cyan, and DMSO-TRCs are yellow. DMSO-TRCs exhibit higher penetration rates than RA-TRCs. Mean±s.e.m.; n>6 fish per group; ≥3 independent experiments. *$p < 0.05$.

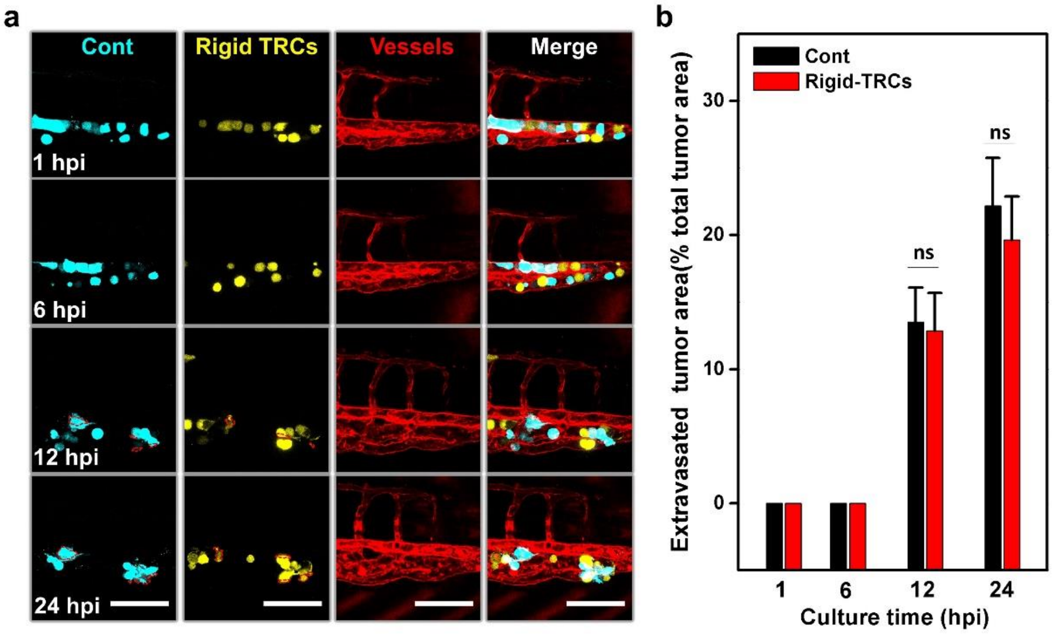


**Supplementary Fig. 23. Rigid TRCs extravasate at a similar rate as control melanoma cells.** (**a**) TRCs were collected and cultured onto rigid plastic for 7 days (Rigid-TRCs) then transfected with pEYFP-N1, and control melanoma cells (Cont) cultured on the rigid plastic were transfected with pECFP-N1. Transfected tumor cells were mixed and co-injected at 1:1 ratio into the cavity of pericardium of 48 hpf embryos. Images in the first two panels show penetration area of control melanoma cells (left panels) and rigid TRCs (right panels) at 1, 6, 12, and 24 hpi respectively, and the next two panels show images of vessels and 3-color overlays. Dashed red lines mark tumor extravasation areas from vessels to surrounding tissues. Scale bars, 100 μm. (**b**) Quantification of extravasated area relative to the total area at the fish tail at different time points: 1, 6, 12, and 24 hpi. Note that there is no extravasation difference between control melanoma cells and rigid TRCs. Color code: Zebrafish blood vessels are red, control melanoma cells are cyan, and rigid TRCs are yellow. Mean±s.e.m.; n=27 larvae; ns = not statistically significant (p=0.86 at 12 hpi; p=0.6 at 24 hpi).

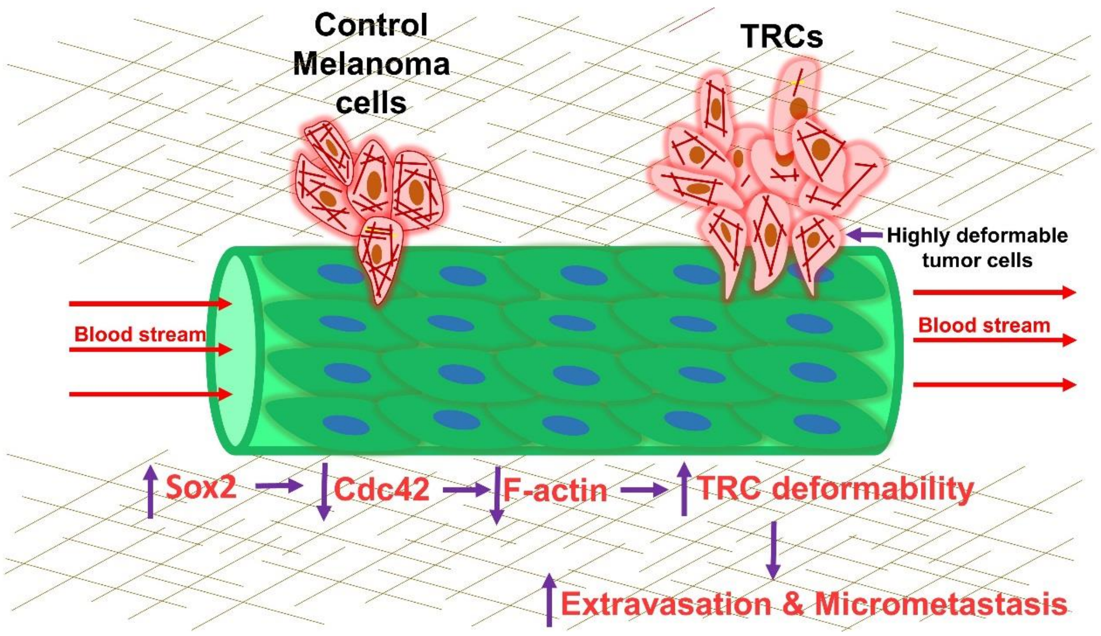


**Supplementary Fig. 24. A working model for extravasation of melanoma cells.** TRCs express high levels of Sox2, downregulating Cdc42, which in turn results in low F-actin and thus high deformability of the cell, leading to high extravasation efficiency and more micrometastasis formation by TRCs, when compared with control melanoma cells. Not drawn to scale.

**Supplementary Movie 1. High resolution extravasation dynamics of TRCs.** TRCs that were anchoring inside blood vessels of the fish tail invaded at a rate of ~2.7 μm per hr into the ECM of peripheral tissues. White arrowheads point to the sites where tumor cells are extravasating. Color code: green, blood vessels; red, tumor cells. Scale bar, 50 μm.

**Supplementary Movie 2. High resolution 3D-reconstruction of TRCs.** Aggregates of TRCs extravasated out of the vessels while some cells were still inside the vessels. This movie is a 3D-reconstruction from part of Fig. 6c at 24 hpi (the bottom right panel). Color code: green, blood vessels; red, tumor cells. Scale bar, 50 μm.
